# Nanomolar Pulse Dipolar EPR Spectroscopy in Proteins; the Cu^II^- Cu^II^ and Nitroxide-Nitroxide Cases

**DOI:** 10.1021/acs.jpcb.1c03666

**Published:** 2021-05-17

**Authors:** Katrin Ackermann, Joshua L. Wort, Bela E. Bode

**Affiliations:** EaStChem School of Chemistry, Biomedical Sciences Research Complex, and Centre of Magnetic Resonance, University of St Andrews, North Haugh, St Andrews, KY16 9ST, Scotland

**Keywords:** EPR spectroscopy, Structural biology, PELDOR/DEER, RIDME, double-histidine motif

## Abstract

The study of ever more complex biomolecular assemblies implicated in human health and disease is facilitated by a suite of complementary biophysical methods. Pulse Dipolar electron paramagnetic resonance Spectroscopy (PDS) is a powerful tool that provides highly precise geometric constraints in frozen solution, however the drive towards PDS at physiologically relevant sub-μM concentrations is limited by the currently achievable concentration sensitivity. Recently, PDS using a combination of nitroxide and Cu^II^ based spin labels allowed measuring 500 nM concentration of a model protein. Using commercial instrumentation and spin labels we demonstrate Cu^II^-Cu^II^ and nitroxide-nitroxide PDS measurements at protein concentrations below previous examples reaching 500 and 100 nM, respectively. These results demonstrate the general feasibility of sub-μM PDS measurements at short to intermediate distances (~1.5 - 3.5 nm), and are of particular relevance for applications where the achievable concentration is limiting.

## Introduction

The study of increasingly complex biomolecular assemblies and their interactions with the cellular environment has driven interest towards holistic structural characterization under conditions with high biological validity. Pulse dipolar EPR spectroscopy (PDS) is a powerful tool for such characterization, and complements X-ray crystallography, NMR, Förster resonance energy transfer (FRET), and cryo-EM data by providing solution-state distance constraints in systems of virtually unlimited size and complexity.^1–9^ Due to these characteristics, PDS is also an emerging technique for conformational studies of protein and nucleic acid complexes *in cellulo*.^10–16^ However, physiological concentrations are often in the sub-μM regime. In combination with low numbers of cells within samples, the challenge is to achieve sufficient absolute sensitivity. Analyzing a representative sample of 61 recent applications of nitroxide- nitroxide pulsed electron-electron double resonance (PELDOR)^17–18^ measurements using the 4- pulse double electron-electron resonance (DEER)^19–20^ sequence reveals the use of spin concentrations between 5 and 400 μM (median 100 μM, mean 116 ± 90 μM, PubMed search on 30 November 2020 for “PELDOR” or “DEER” and “EPR” covering 2017 to 2020, see Supporting Information (SI) for more details) demonstrating the current state of the art. While measurements down to 1 μM should be feasible we could not identify a single published example. Recently, Cu^II^-nitroxide 5-pulse relaxation induced dipolar modulation enhancement (RIDME)^21–22^ measurements at 500 nM concentration in a protein *in vitro* allowed not only precise distance measurements but also determination of the binding affinity.^23^ Thereby, demonstrating the high-affinity of genetically encoded double-histidine motifs to Cu^II^ ions,^24–25^ and their suitability as labelling sites for low concentration studies.^26^

**Figure 1:**
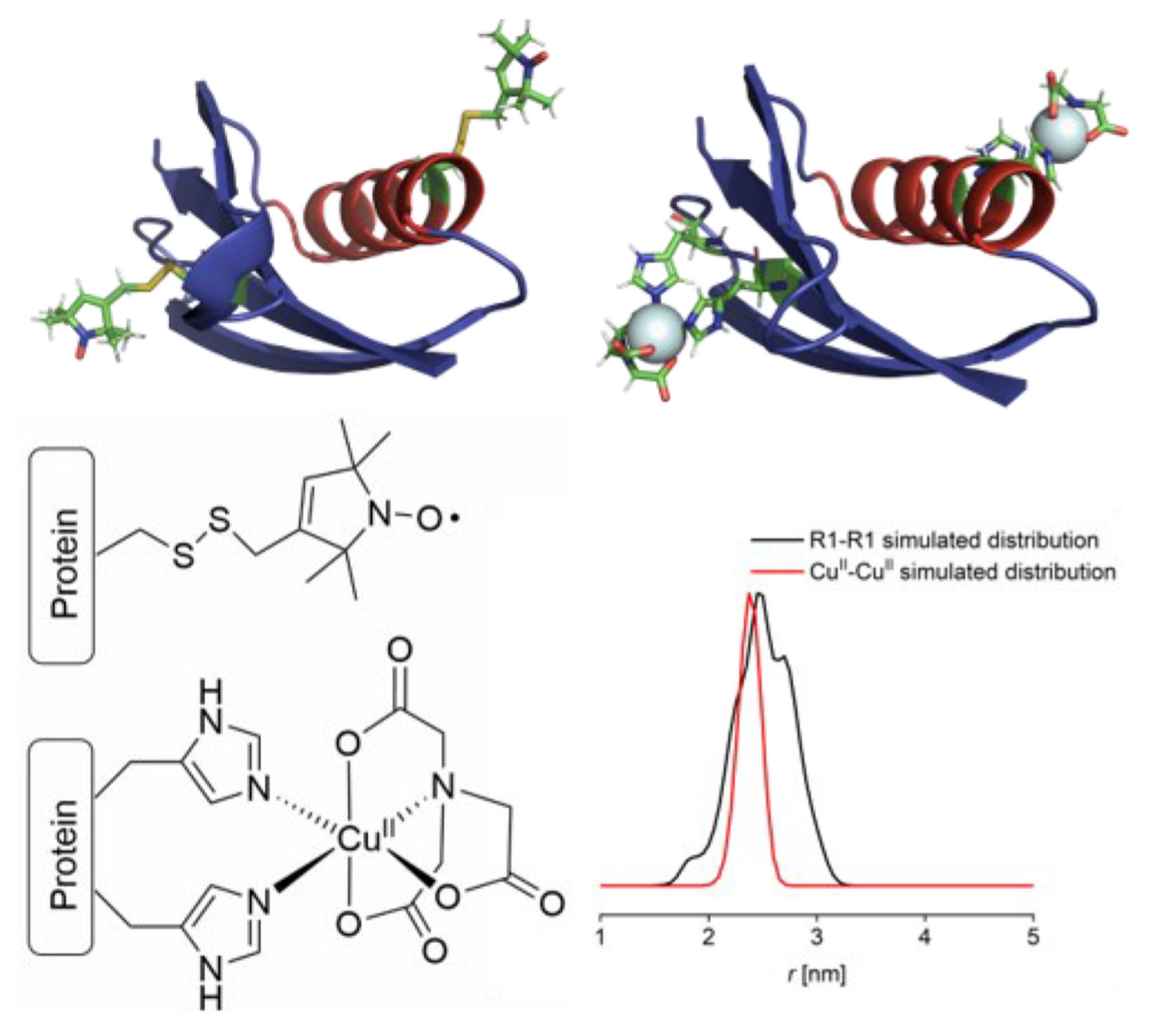
GB1 constructs, spin label structures and simulated distance distributions. Top: Cartoon representations of GB1 constructs I6R1/K28R1 (left) and I6H/N8H/K28H/Q32H (right), with spin labels shown in stick representation. Bottom: Chemical structures of R1 nitroxide and double histidine Cu^II^-NTA spin labels (left). Corresponding simulated distance distribution (right) for each construct, shown in black and red, respectively.

Herein, we approach practical concentration limits associated with PDS experiments and found Cu^II^-Cu^II^ RIDME measurements and nitroxide-nitroxide PELDOR measurements feasible at 500 nM and 100 nM protein concentration, respectively (corresponding to spin concentrations of 1.6 μM and 200 nM, respectively). Importantly, these measurements were performed in a commercial non-broadband Q-band spectrometer, using well-established spin labels, methanethiosulfonate (MTSL)^27–28^ and Cu^II^-nitrilotriacetic acid (Cu^II^-NTA)^25^ (figure 1). PDS measurements in a biological system down to 100 nM protein concentration for nitroxide-nitroxide DEER and 500 nM for Cu^II^-Cu^II^ RIDME are unprecedented.

Commercial instruments have been used successfully for PELDOR measurements at low μM concentration.^29^ Concentration sensitivity has been demonstrated to further improve in homebuilt high-power resonator-free spectrometers^30–31^ or by implementation of arbitrary waveform generators (AWGs) and shaped pulses that yield higher spin inversion efficiencies.^32–36^ Additionally, novel pulse sequences have shown to enhance measurement sensitivity.^37–40^ Trityl- based radicals^41–43^ with exquisitely narrow spectral linewidths have been measured at 45 nM protein (90 nM spin) concentration^41^ employing the single-frequency double quantum coherence (DQC)^44^ experiment. This is a remarkable achievement owed to bespoke narrow line spin labels allowing use of a single-frequency technique. It is not currently established where the limits are for the most common 4-pulse DEER method applied to the most popular nitroxide labels, nor for the emerging use of RIDME on Cu^II^-labels.

## Experimental Methods

### Protein expression and purification

#### Constructs

For this study, two constructs of the immunoglobulin-binding B1 domain of group G streptococcal protein G (GB1) were used: a tetra-histidine mutant with two double-histidine Cu^II^- binding motifs (I6H/N8H/K28H/Q32H) and a double-cysteine mutant (I6C/K28C), both have been described previously.^24^ The first construct was obtained as a kind gift from Prof. Saxena, the second construct was bought from GenScript, and the pET-11a vector was used for both constructs.

#### Expression and purification

Both GB1 constructs were expressed and purified according to the procedure described in Wort *et al*.^23^ MTSL labelling of the double-cysteine construct was performed as described.^23^ Spin labelling was confirmed via MALDI-TOF using the in-house mass spectrometry facility, and labelling efficiencies obtained from continuous wave (CW) EPR spectra were ≥ 89 % compared to MTSL as a standard.^23^

### Electron paramagnetic resonance (EPR) spectroscopy

Samples for pulse EPR measurements were prepared with varying protein and Cu^II^-NTA concentrations in deuterated gel filtration buffer and 50% (v/v) deuterated ethylene glycol (Deutero) was used for cryoprotection as previously described.^23^ The samples with a final volume of 65 μL were transferred to 3 mm quartz EPR tubes which were immediately frozen in liquid nitrogen.

Pulse EPR experiments were performed at Q-band frequency (34 GHz) operating on a Bruker ELEXSYS E580 spectrometer with a 3 mm cylindrical resonator (ER 5106QT-2w in TE012 mode). Pulses were amplified by a pulse travelling wave tube (TWT) amplifier (Applied Systems Engineering) with nominal output of 150 W. Temperature was controlled via a cryogen-free variable temperature cryostat (Cryogenic Ltd) operating in the 3.5 to 300 K temperature range.

Temperature optimization for pulse EPR measurements were performed between 10 K and 50 K. Longitudinal relaxation times (*T*_1_) were estimated from 3-pulse inversion recovery measurements under the mono- or bi-exponential approximation. Transverse dephasing times (*T*_m_) were estimated from 2-pulse electron-spin echo decay measurements, data was fitted to a stretched exponential function, with the stretching exponent varying from 1-2. Subsequently, relaxation times were used in downstream processing to determine relative sensitivities per temperature as described previously.^23^

Instantaneous diffusion (ID, also referred to as dipolar dephasing) was qualitatively assessed by measuring Hahn echo decays with varying flip angles of the second pulse as described.^45^

#### Pulse dipolar EPR

PELDOR and RIDME measurements were obtained at Q-band frequency (34 GHz) using a second frequency option (E580-400U).

PELDOR experiments were performed with the 4-pulse DEER^18, 20, 46^ pulse sequence (π/2(vA) – τ1 – π(vA) – (τ1 + t) – π(vB) – (τ2 - t) – π(vA) – τ2 – echo) at 50 K as described previously,^47^ with a frequency offset (pump – detection frequency) of +80 MHz (~3 mT). Shot repetition times (SRT) were set to 4 or 5 ms; τ_1_ was set to 380 ns, and τ_2_ was set to 1500 ns for the 100 and 500 nM samples (recorded for 47 and 14.5 hours, respectively) and to 3000 ns for the 25 μM sample (recorded for ~70 minutes) of the double-cysteine construct spin-labelled with MTSL (I6R1/K28R1). Pulse lengths were 16 and 32 ns for π/2 and π detection, and 12 or 14 ns for the ELDOR π pump pulse for the micromolar or nanomolar samples, respectively. The pump pulse was placed on the resonance frequency of the resonator and applied to the maximum of the nitroxide field-swept spectrum.

5-pulse RIDME experiments^22^ were recorded with the pulse sequence (π/2 – τ1 – π – (τ1 + t) – π/2 – T_mix_ – π/2 – (τ2 - t) –π – τ2 – echo) at 30 K at the field position corresponding to the maximum of the Cu^II^ field-swept spectrum with 8-step phase cycling, a τ1 of 400 ns, SRT of 407 μs, and a critically coupled resonator (high Q). The 500 nM sample of the tetra-histidine construct (I6H/N8H/K28H/Q32H) was recorded for 63.5 hours with a mixing time of 34 μs, while the 500 μM sample was recorded for ~15 minutes at three different mixing times (5, 35, and 65 μs, 1 scan) to facilitate the removal of artefacts or residual ESEEM by deconvolution.^23^

“Dummy” PELDOR and RIDME measurements (1 scan each) were recorded at 500 nM and 25 μM protein concentration for sensitivity estimates as described.^23^ Briefly, in these measurements, all delays are set to be constant, shifting the entire sequence by the dipolar increment, thus yielding a trace that is only varied by thermal noise on the acquired echo. RIDME experiments were recorded both, with a critically coupled and an over-coupled resonator to determine the effect of high versus low Q on sensitivity.

PDS experiments were analyzed using DeerAnalysis2015.^48^ PELDOR data were first background-corrected using a 3-dimensional homogeneous background function before Tikhonov regularization followed by statistical analysis using the validation tool in DeerAnalysis2015, varying background start from 5 to 80% of the trace length in 16 trials. Resulting background start time for the best fit was then used as starting point for a second round of Tikhonov regularization followed by a second round of statistical analysis, this time also including the addition of 50% random noise in 50 trials. For the 100 nM and 500 nM I6R1/K28R1 sample the first validation rounds resulted in a best fit where the background fit had a positive and thus unphysiological slope; therefore, data were cut iteratively by 10% and then by 20% of the initial trace length and Tikhonov regularization and validation were repeated. Since cutting of the data did not afford a falling background function while leaving the resulting distributions essentially unchanged, the full-length data were used for further processing.

RIDME data were first background-corrected using a homogeneous 6-dimensional background function before Tikhonov regularization followed by statistical analysis varying background start from 5 to 30% of the trace length in 8 trials and varying the background dimension from 3 to 6 in 7 trials. Resulting background start time and dimension for the best fit were then used as starting points for a second round of Tikhonov regularization followed by a second round of statistical analysis, this time also including the addition of 50% random noise in 16 trials. Validation trials from the second validation round for PELDOR and RIDME data were pruned with a prune level of 1.15, where trials exceeding the root mean square deviation of the best fit by at least 15% are discarded. In all cases the regularization parameter α was chosen according to the L-curve criterion^49^ and the goodness-of-fit. RIDME data obtained at 500 μM protein concentration were cut at a trace length of 2200 ns to improve estimation of the background function and fitting.

### Simulations and Modelling

Distance distributions were modelled based on the I6H/N8H/K28H/Q32H construct (PDB ID: 4WH4);^24^ all modelling was performed using MMM 2018.^50–51^ For the I6R1/K28R1 construct, histidine residues at positions 6 and 28 of the I6H/N8H/K28H/Q32H construct were mutated to cysteine residues, while histidine residues at positions 8 and 32 were mutated to asparagine and glutamine residues, and R1 moieties were introduced at residues 6 and 28 in MMM 2018. Labelling with either MTSL or Cu^II^-NTA was modelled under ambient temperature (298 K), and the corresponding predicted distance distributions are shown in the main text. For the Cu^II^-NTA labelling, a correction factor of 1.13 was applied to the distance axis to correct for the g-value change from a free electron to bound Cu^II^, assuming a g_iso_ of 2.13 for bound Cu^II^-NTA.^52^

Cartoon structural representations of I6H/N8H/K28H/Q32H and I6R1/28H/32H GB1 constructs were generated using Pymol^53^ and MTSL wizard,^54^ respectively.

## Results and Discussion

**Figure 2:**
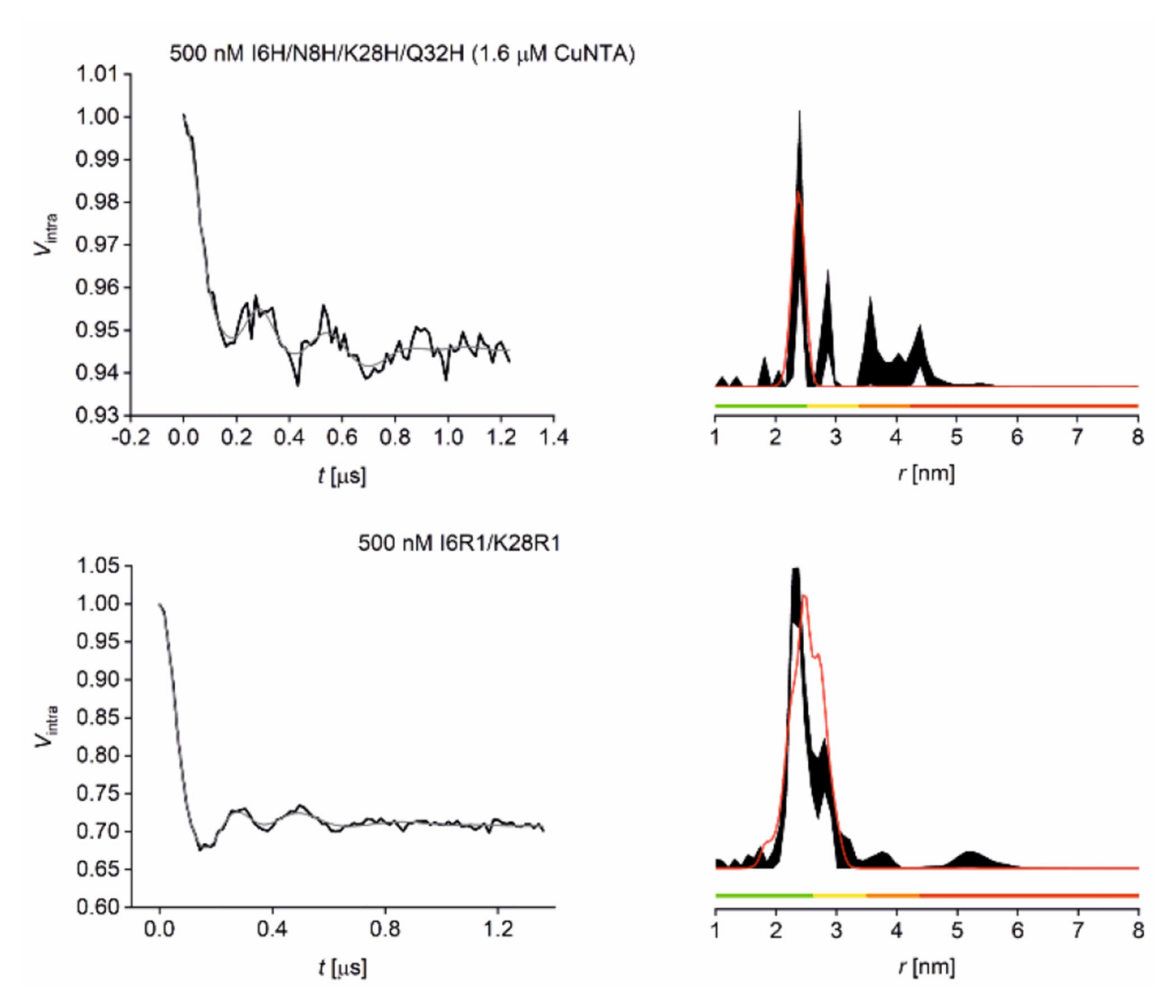
500 nM GB1 Cu^II^-Cu^II^ RIDME and nitroxide-nitroxide PELDOR Q-band data at 30 and 50 K, respectively. Top: RIDME data for 500 nM GB1 tetra-histidine with 1.6 μM Cu^II^-NTA added. Bottom: PELDOR data for 500 nM GB1 I6R1/K28R1. Left: Background-corrected data (black) and fit (grey). Right: Corresponding distance distributions given as 95% confidence intervals (± 2σ) with 50% noise added for error estimation during statistical analysis; simulated distance distributions are shown in red. Color bars represent reliability ranges (green: shape reliable; yellow: mean and width reliable; orange: mean reliable; red: no quantification possible).

In the current study, *Streptococcus sp*. Group G protein G, B1 domain (GB1) constructs (I6R1/K28R1 and I6H/N8H/K28H/Q32H) were used as biological model systems (figure 1). GB1 has been used extensively in previous EPR methodology studies.^23–26, 55–58^ We have shown previously that nitroxide-detected Cu^II^-nitroxide and Cu^II^-Cu^II^ RIDME are similar in sensitivity and roughly two orders of magnitude more sensitive than Cu^II^-Cu^II^ PELDOR when limited to rectangular pulses.^23^ Here, we endeavored to test the sensitivity of the most widespread pulse dipolar EPR methodology, nitroxide-nitroxide PELDOR.^59^ Therefore Cu^II^-Cu^II^ RIDME and nitroxide-nitroxide PELDOR were measured at 500 nM concentration for a direct comparison of experiment sensitivity (figure 2).

The optimum temperatures with respect to sensitivity were found to be 30 K and 50 K, respectively (see SI). As RIDME is a single frequency technique, it can be performed with all pulses coinciding with the resonance frequency of the resonator and thus benefits in sensitivity compared to double frequency techniques, such as the 4-pulse DEER sequence where detection is generally performed off-resonance. This sensitivity gain in dependence of the cavity quality factor being adjusted to meet the required bandwidth could be quantified as approximately a factor 2 (see SI). Furthermore, the influence of instantaneous diffusion (that occurs when dephasing is induced by dipolarly coupled spins being inverted by detection pulses reducing the detected echo) was shown to be negligible in the I6R1/K28R1 construct at both 500 nM and 25 μM concentrations (see SI).

**Figure 3:**
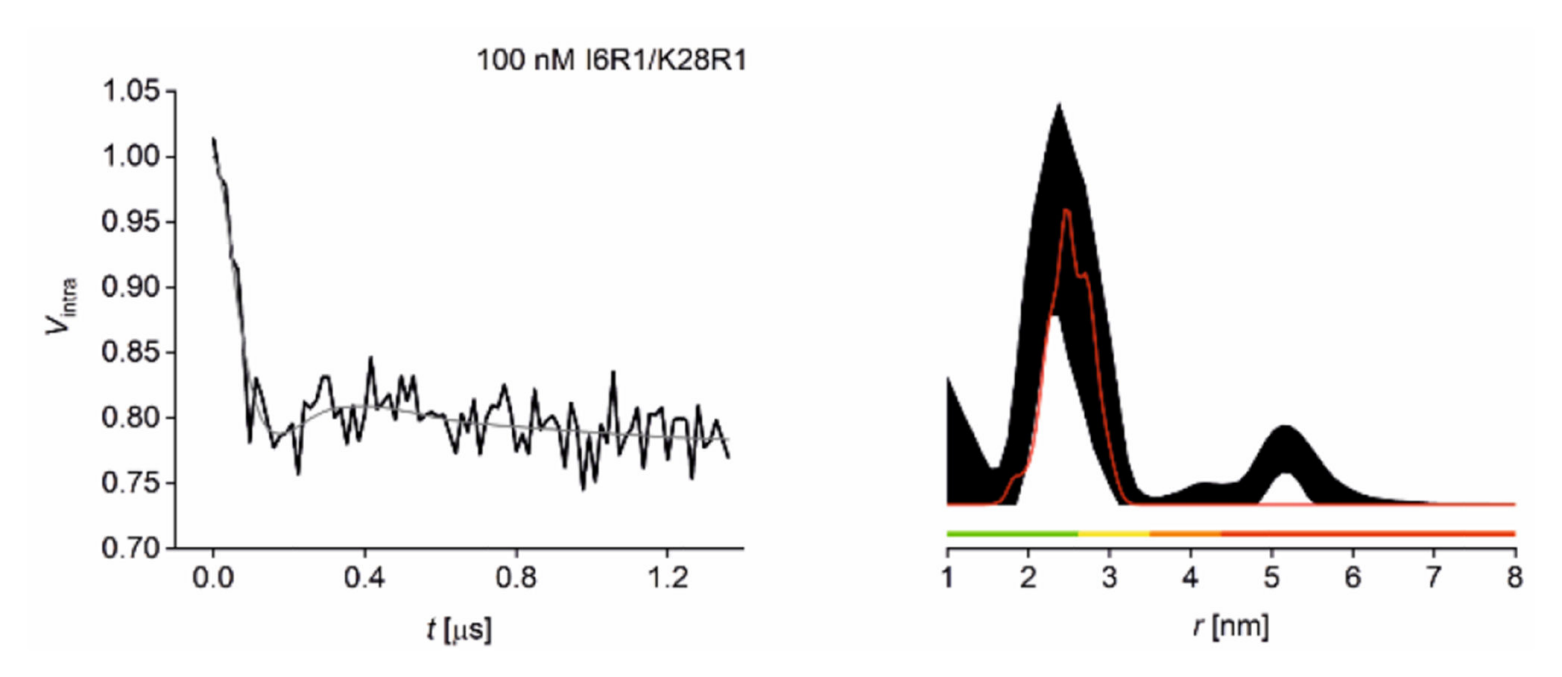
100 nM GB1 Q-band PELDOR data at 50K. Left: Background-corrected PELDOR data (black) and fit (grey) for 100 nM I6R1/K28R1 GB1. Right: Corresponding distance distribution given as 95% confidence intervals (± 2σ) with 50% noise added for error estimation during statistical analysis; simulated distance distributions are shown in red. Color bars represent reliability ranges (green: shape reliable; yellow: mean and width reliable; orange: mean reliable; red: no quantification possible).

For the Cu^II^-Cu^II^ RIDME data shown in figure 2, only the distance peak at ~2.5 nm was shown to be stable upon data validation. Additional measurements at 500 μM protein concentration suggested the distribution peaks above 2.5 nm were artefacts, insignificant in the 95% confidence interval (see SI). This indicated that measurements at 500 nM tetra-histidine protein concentration likely approached the lower concentration limit for Cu^II^-Cu^II^ RIDME in our hands. It should be noted that the poor modulation depth (5.5%) is a result of the limiting affinity of Cu^II^-NTA for the β-sheet double histidine motif.^23^ Pulse dipolar EPR methods allow precise determination of binding affinities from PELDOR^60–62^ and RIDME^63^ data. The observed modulation depth is consistent with predictions using binding affinities previously derived from Cu^II^-nitroxide RIDME pseudo-titration^23^ and extrapolated ITC data (see SI). Conversely, for the nitroxide-nitroxide PELDOR data the bimodal distribution shown in figure 2 was recapitulated in additional measurements at 25 μM I6R1/K28R1 protein concentration (see SI). This suggested that measurements at 500 nM protein concentration were not yet testing the lower concentration limit for nitroxide-nitroxide PELDOR. To test this hypothesis, nitroxide-nitroxide PELDOR was also measured at 100 nM protein (200 nM spin) concentration (figure 3).

The nitroxide-nitroxide PELDOR data shown in figure 3, measured with the same dipolar evolution time for comparison, indicate that at 100 nM the retrieved experimental distribution is no longer bimodal, however the mean distance is still retrieved as the only significant peak following data validation. Nevertheless, the relatively poor signal-to-noise ratio mandates a regularization parameter that does not allow resolving both distance populations (SI). This loss in resolution has been confirmed using other processing approaches (see SI for details). This experiment thus highlights the dependence of distance resolution on achievable signal-to-noise and thus, on spin concentration. Together, this suggests that 100 nM approaches the minimum concentration achievable for reliable determination of the mean distance from nitroxide-nitroxide PELDOR under our conditions; it is already beyond a reliable determination of the distance distribution shape. Sensitivity analysis shows that measurement of I6R1/K28R1 at 100 nM is a factor ~15 noisier than measurement at 500 nM, rather than the factor 5 expected from the concentration difference. The additional factor 3 can be considered a penalty for the challenging measurement optimization at these very low concentrations (see SI).

Comparing the relative sensitivities of nitroxide-nitroxide PELDOR and Cu^II^-Cu^II^ RIDME reveals the former to be approximately 10-fold more sensitive (see SI). The 3 main factors contributing to the relative sensitivity are echo amplitude (i.e., signal-to-noise), modulation depth, and averaging rate. For Cu^II^-Cu^II^ RIDME the smaller signal is largely compensated by the faster averaging. However, the modulation depth is a limiting factor for sensitivity. This is to be expected for a non-covalent spin-label with dissociation constants in the high nM (α-helix) and low μM (β-sheet) regime. Overcoming the low modulation depth will make sensitivity of Cu^II^-Cu^II^ RIDME competitive. Simulation of a tetra-histidine construct containing a pair of α-helical binding sites reveals modulation depths > 12% for Cu^II^-Cu^II^ RIDME under otherwise identical conditions (see SI). Possible strategies to further improve modulation depths include insertion of an artificial amino-acid bearing a covalent Cu^II^ centre to overcome the limiting equilibrium constants,^64^ or measuring nitroxide detected Cu^II^-nitroxide RIDME in excess Cu^II^ chelate spin label. This will shift the binding equilibrium into saturation of the binding site and achieve modulation depths approaching 50%, independent of protein concentration.

Comparing nitroxide-nitroxide PELDOR with available Cu^II^-nitroxide RIDME data^23^ suggests the latter to be an additional factor ~1.5 more sensitive potentially allowing measurements even below 100 nM protein concentration (see SI). Additionally, RIDME measurements may be less prone to the optimization penalty found for 4-pulse DEER at 100 nM as the single-frequency method will need fewer parameters to be set. These findings showcase that in favorable circumstances superb concentration sensitivities are achievable using commercial instrumentation and spin labels. In the case of the widely applied nitroxide-nitroxide 4-pulse DEER experiment, concentration sensitivities orders of magnitude greater than routinely applied (≥10 μM) are possible using rectangular pulses at Q-band frequencies. Additionally, Cu^II^-Cu^II^ RIDME measurements showcase that systems not amenable to conventional thiol-based covalent spin labelling are also accessible in the sub-μM concentration regime, when used in conjunction with double-histidine motifs. Concentrations realized here for Cu^II^-Cu^II^ RIDME are more than an order of magnitude below published applications of the 5-pulse RIDME experiment on metal-metal spin systems (≥ 25 μM; see SI).

Nevertheless, at these low concentrations long distances or more complex distance distributions will be a challenge for both, nitroxide-nitroxide DEER and Cu^II^-Cu^II^ RIDME experiments. The presence of conformational flexibility or two (or more) conformational states will result in broad or multimodal distance distributions, respectively. The superposition of frequencies will lead to less pronounced oscillations and a larger ambiguity in the distance analysis.^65^ While the distance distribution mean and width were recoverable from the 100 nM GB1 sample the bimodal distribution was only resolved in the 500 nM sample. Long distances and highly resolved distance distributions require longer dipolar evolution times. In these cases, sensitivity enhancement is achievable by full deuteration of protein and matrix due to decreased echo dephasing (increased phase memory time *T*_m_).^66–67^ The benefit that can be realised from deuteration will strongly depend on the required trace lengths.

## Conclusions

Benchmarking the nanomolar sensitivity is truly promising as a pathway to novel applications and may facilitate study of systems previously thought to be beyond the scope of pulse EPR spectroscopy. Perhaps most importantly, our results emphasize that commercial instrumentation and standard labelling protocols already yield sufficient concentration sensitivity for applications in the sub-μM regime. Indeed, a very recent study published after our initial report^68^ has demonstrated DEER distance measurements *in cell* for Gd^III^ at ~350 nM protein concentrations and *in vitro* for nitroxides down to 120 nM protein / 200 nM spin concentration.^69^ The imminent adoption of cryogenically cooled preamplifiers for EPR spectroscopy^70^ heralds a further order of magnitude of sensitivity improvement. Embracing the opportunity to measure at concentrations two to three orders of magnitude below past practice will bring new science into reach that is currently sample limited in either concentration or absolute amount.

## Open data statement

The research data supporting this publication can be accessed at https://doi.org/10.17630/6ca0de75-904d-4768-8a21-2c235a717412.^71^

## Supplementary Material

supplementary information
