# Supplementary material for: Nanomolar Pulse Dipolar EPR Spectroscopy in Proteins; the CuII- CuII and Nitroxide-Nitroxide Cases
Source: J Phys Chem B. Author manuscript; Available in PMC 2021 Jun 24. (PMC7611071; doi:10.1021/acs.jpcb.1c03666)
Supplement: supplementary information [file EMS124570-supplement-supplementary_information.pdf]

Nanomolar Pulse Dipolar EPR Spectroscopy in Proteins; the Cu^{II}- Cu^{II} and Nitroxide-Nitroxide Cases.

Katrin Ackermann, Joshua L. Wort, Bela E. Bode*

EaStCHEM School of Chemistry, Biomedical Sciences Research Complex and Centre of Magnetic Resonance, University of St Andrews, St Andrews, KY16 9ST, Scotland

Supporting Information

Content

1. Temperature-dependent relaxation behavior page S2
2. Additional RIDME data (I6H/N8H/K28H/Q32H construct) page S4
3. Additional PELDOR data (I6R1/K28R1 construct) page S6
4. Deep neural network processing and wavelet denoising page S8
5. Instantaneous diffusion page S11
6. Sensitivity considerations page S12
7. Literature search page S15
8. References page S16

1. Temperature-dependent relaxation behavior

Relaxation measurements performed at different temperatures from 10 K to 50 K informed about the temperature with optimum sensitivity for the NO-NO PELDOR and the Cu^{II}-Cu^{II} RIDME experiments, respectively.

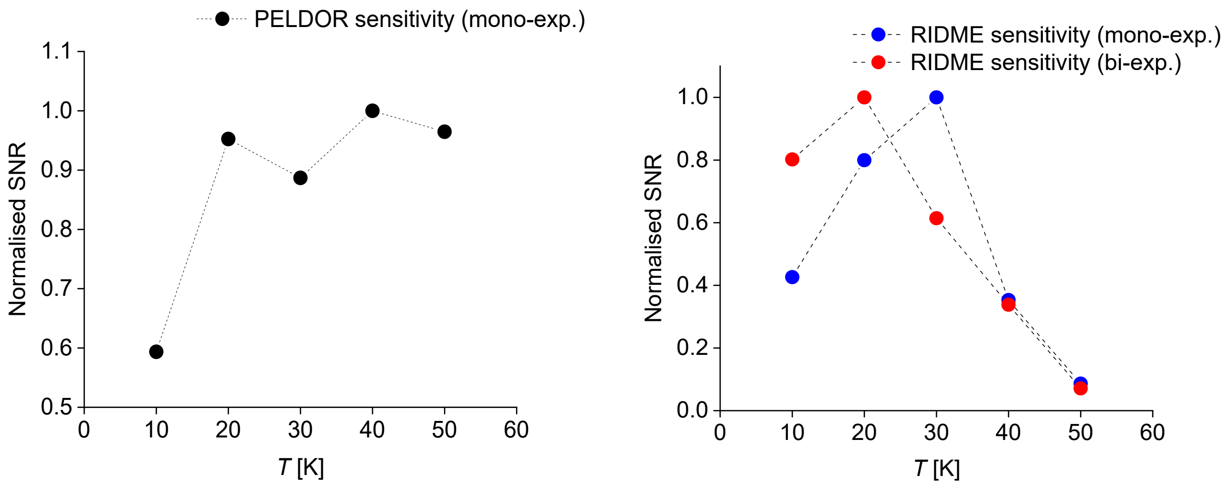

Figure S1: Sensitivity profiles for nitroxide-nitroxide PELDOR (left, mono-exponential approximation of T_1) and Cu^{II}-Cu^{II} RIDME (right, mono- and bi-exponential approximation of T_1 in red and blue, respectively). Profiles were calculated using equations given in Wort et al.,[1] and measurement temperatures of 50 K and 30 K (for the mono-exponential approximation of T_1) show > 90% of maximal sensitivity under experiment conditions. SNR = signal-to-noise ratio.

The longitudinal relaxation times (T_1) estimated under the mono- and bi-exponential approximations, 1/e times, and 1/2e^2 times for Cu^{II}-chelate and R1 nitroxide are given in tables S1-2, respectively. Note that at all temperatures, the 1/2e^2 times are within 20% of the 1/e times, indicating that the relaxation behavior is well met by the mono-exponential approximation. The phase memory times (T_m) estimated under the stretched exponential approximation for Cu^{II}-chelate and R1 nitroxide are given in tables S3-4, respectively.

**Temperature**	**Mono-exponential T_1**	**Bi-exponential T_1**	**1/e time**	**1/2e^2 time**
10 K	4800 µs	1500 µs (0.23) / 6130 µs (0.77)	5201 µs	5241 µs
20 K	234 µs	134 µs (0.44) / 334 µs (0.56)	301.0 µs	286.7 µs
30 K	52.8 µs	31.0 µs (0.66) / 97.8 µs (0.54)	61.5 µs	57.5 µs
40 K	15.6 µs	11.2 µs (0.68) / 25.3 µs (0.32)	19.6 µs	18.5 µs
50 K	7.6 µs	5.6 µs (0.81) / 15.6 µs (0.19)	9.2 µs	8.6 µs

Table S1: Estimated T_1 values under mono- and bi-exponential approximations for Cu^{II}-chelate.

Temperature	Mono-exponential T_1	Bi-exponential T_1	1/e time	1/2e^2 time
10 K	397 ms	196 ms (0.23) / 486 ms (0.77)	372 ms	302 ms
20 K	39.8 ms	11.7 ms (0.25) / 53.5 ms (0.75)	52.0 ms	47.3 ms
30 K	8.1 ms	2.1 ms (0.13) / 9.6 ms (0.87)	17.7 ms	17.5 ms
40 K	4.2 ms	2.1 ms (0.42) / 6.5 ms (0.58)	5.7 ms	5.3 ms
50 K	2.2 ms	1.2 ms (0.52) / 3.9 ms (0.48)	3.1 ms	3.0 ms

Table S2: Estimated T_1 values under mono- and bi-exponential approximations for R1 nitroxide.

Temperature	Stretched Exponential *T*m	Stretching Exponent
10 K	6.8 µs	1.17
20 K	6.2 µs	1.19
30 K	5.9 µs	1.45
40 K	3.7 µs	1.10
50 K	2.6 µs	1.07

Table S3: Estimated T_m values under the stretched exponential approximation for Cu^{II}-chelate.

Temperature	Stretched Exponential *T*m	Stretching Exponent
10 K	9.1 µs	1.58
20 K	9.2 µs	1.63
30 K	7.0 µs	1.23
40 K	7.3 µs	1.45
50 K	6.8 µs	1.26

Table S4: Estimated T_m values under the stretched exponential approximation for R1 nitroxide.

2. Additional RIDME data (I6H/N8H/K28H/Q32H construct)

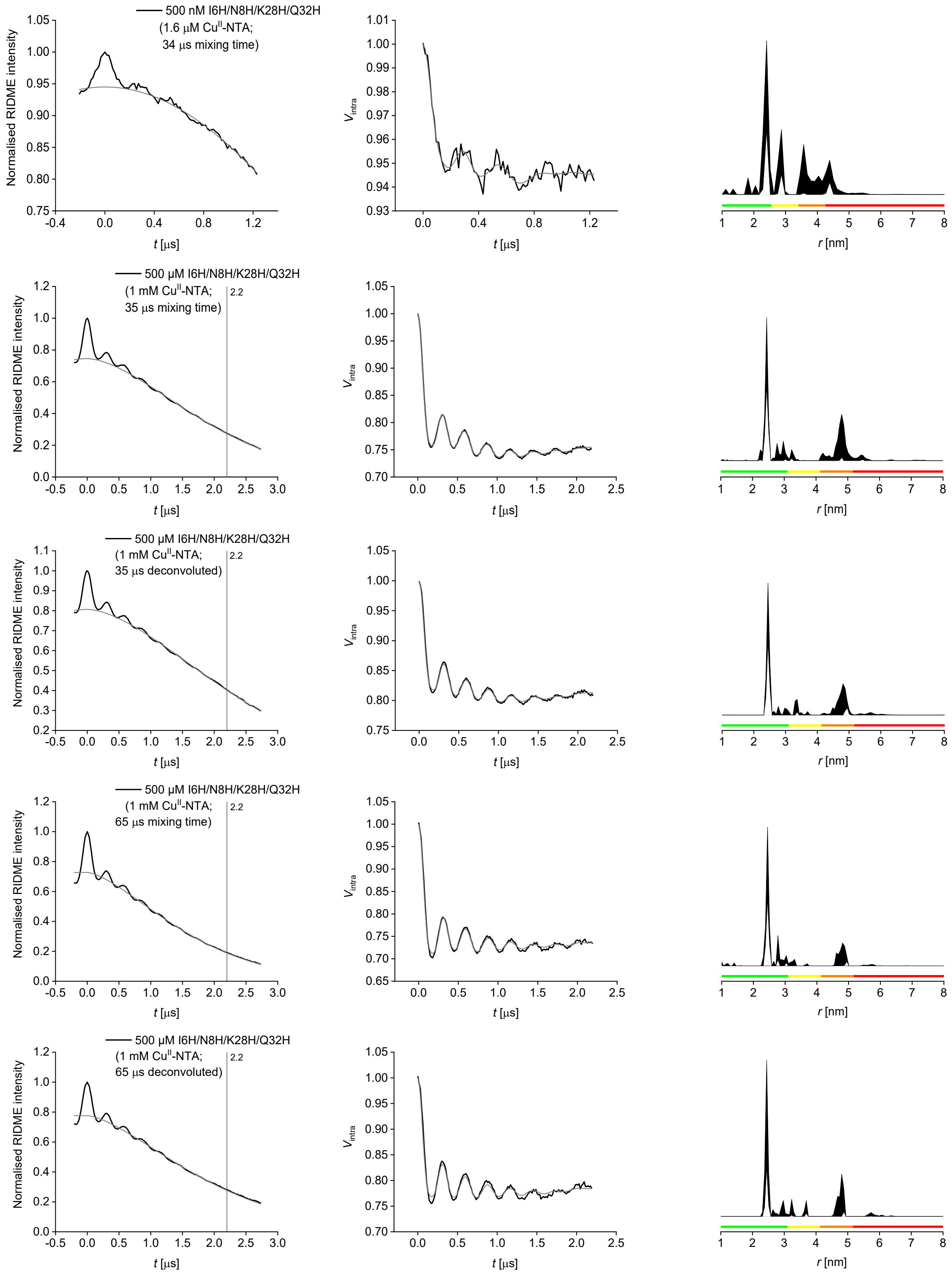

Figure S2: Comparison of RIDME data obtained at different concentrations and processing conditions. Top: 500 nM, measurement with 34 μs mixing time, all other rows: 500 μM protein, mixing times in μs are indicated (35 or 65 μs, or deconvoluted, corresponding to the ratios 35/5 or 65/5, respectively). Left: raw RIDME traces (black) with background function (grey), the vertical line indicates where traces were cut; middle: background-corrected data (black) with fit (grey); right: corresponding distance distributions given as 95% confidence intervals (± 2σ) with 50% noise added for error estimation during statistical analysis. Color bars represent reliability ranges (green: shape reliable; yellow: mean and width reliable; orange: mean reliable: red: no quantification possible).

Additional RIDME measurements were performed at high protein concentration to assess the unexpected ~3 nm peak observed in the 500 nM sample of the tetra-histidine construct. This peak is not present at the higher concentration sample measured using different mixing times for deconvolution and recorded with approximately two-fold longer evolution time at high SNR. This results in a more accurate estimation of the background function and allows the data to be cut, which subsequently improves fitting.
This is not feasible at the lower concentration; however, we can demonstrate that the peak at the expected distance of ~2.4 nm is stable while the additional peaks are fully uncertain after statistical analysis (i.e., are dependent on the choice of background start time, zero-time artefact, etc.).
The figure below shows the 500 nM sample of the tetra-histidine construct with different choices of background start time, demonstrating the effect on the additional peaks while the 'true' distance peak is unaffected.

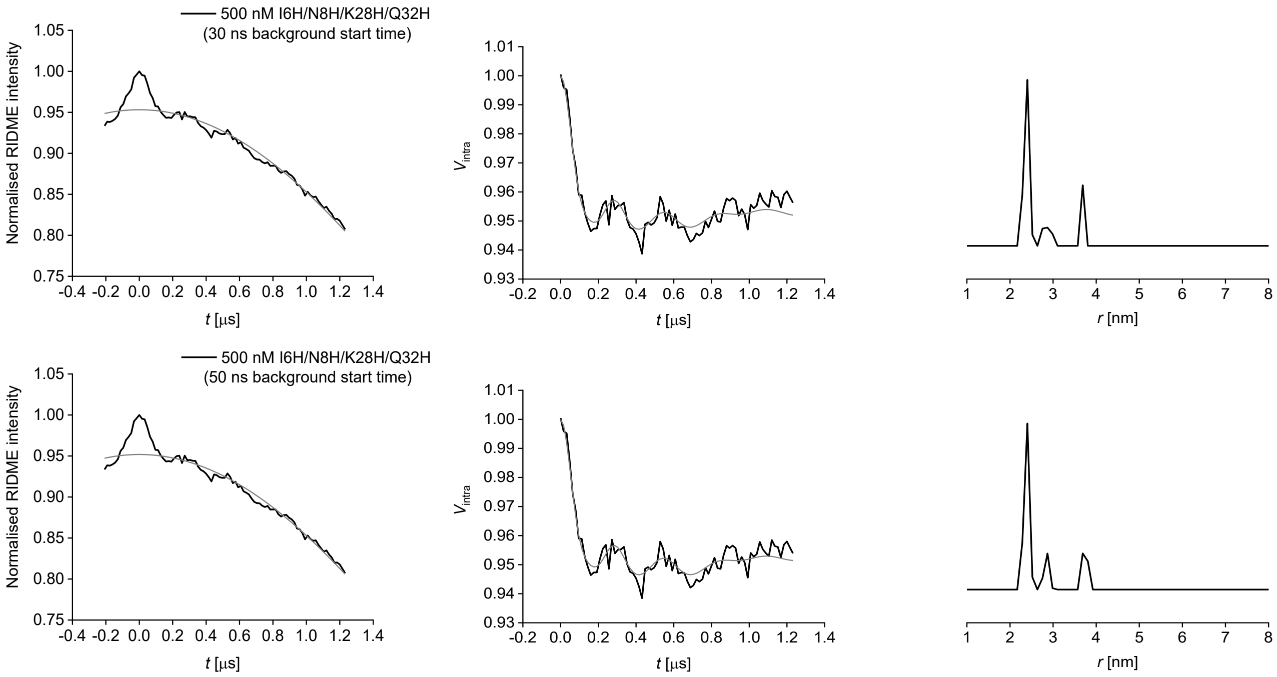

Figure S3: Comparison of RIDME data obtained at 500 nM protein concentration. The same experimental data as shown in Figure S2 top row was processed with either a background start time of 30 ns (top) or 50 ns (bottom). Left: raw RIDME traces (black) with background function (grey); middle: background-corrected data (black) with fit (grey); right: corresponding distance distributions. At the earliest background start time the ~3 nm peak is mostly suppressed, while already at 50 ns start time it is clearly visible as a distinct peak.

3. Additional PELDOR data (I6R1/K28R1 construct)

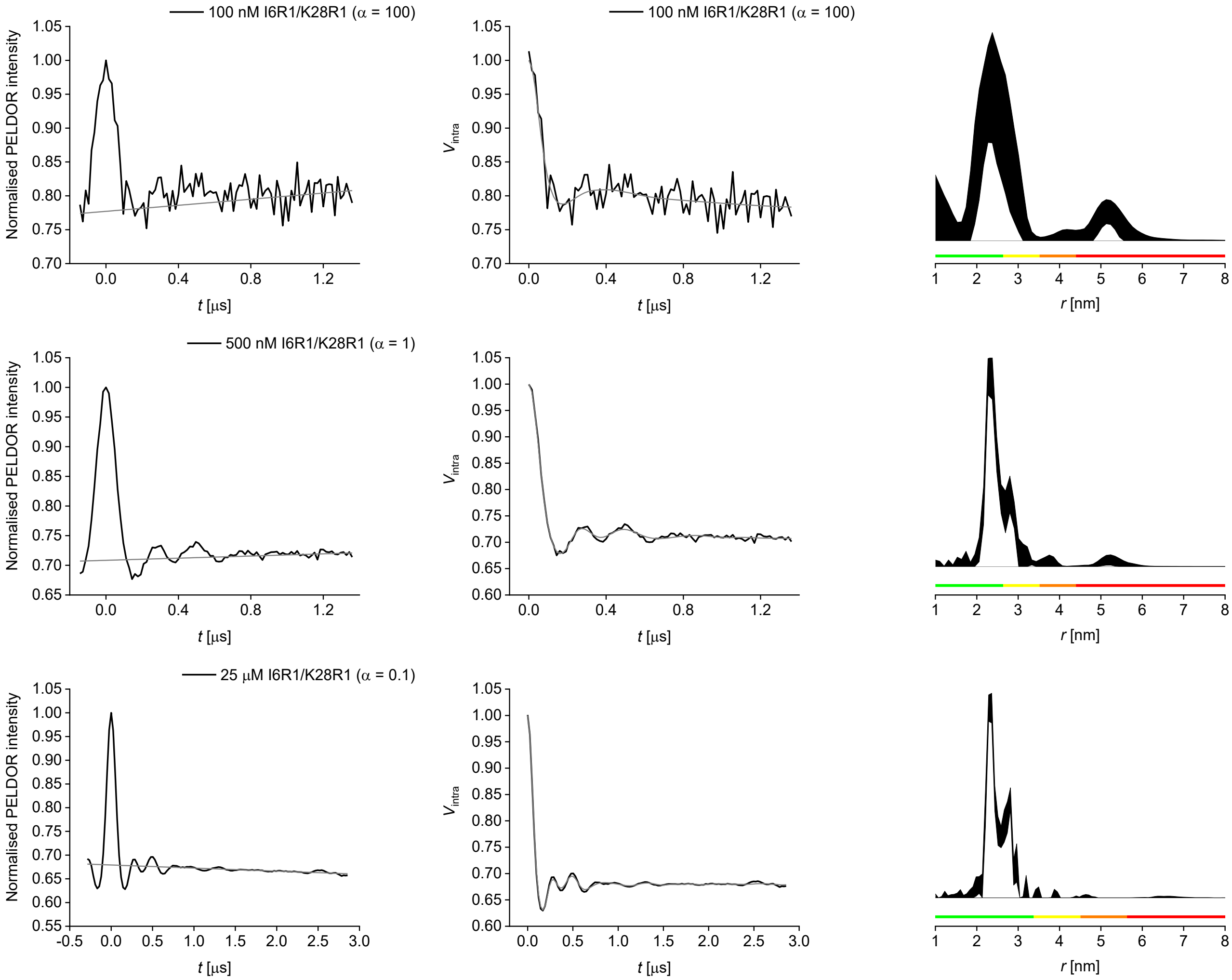

Figure S4: Comparison of PELDOR data obtained at different concentrations. Top: 100 nM, middle: 500 nM, bottom: 25 μM protein. Left: raw PELDOR trace (black) with background function (grey); middle: background-corrected data (black) with fit (grey); right: corresponding distance distributions given as 95% confidence intervals (± 2σ) with 50% noise added for error estimation during statistical analysis. Color bars represent reliability ranges (green: shape reliable; yellow: mean and width reliable; orange: mean reliable: red: no quantification possible). Regularization parameters varied depending on concentration: 100 for 100 nM, 1 for 500 nM, and 0.1 for 25 μM protein.

At 25 μM protein concentration of the I6R1/K28R1 construct the trace can be recorded over a longer evolution time, thus improving reliability for longer distances in the corresponding distribution. These data indicate that the bimodal shape of the distribution peak at 2.5 nm is indeed true. This suggests that down to concentrations as low as 500 nM one can reliably retain the true distance information. At even lower concentration (100 nM) the mean distance can still be retrieved; however, the resolution of the bimodality is lost due to i) low SNR and ii) necessity of a larger regularization parameter.

To confirm this observation, data for the 500 nM were processed with a regularization parameter of 10 and 100, and vice versa, the 100 nM data were processed with smaller regularization parameters, as shown below.

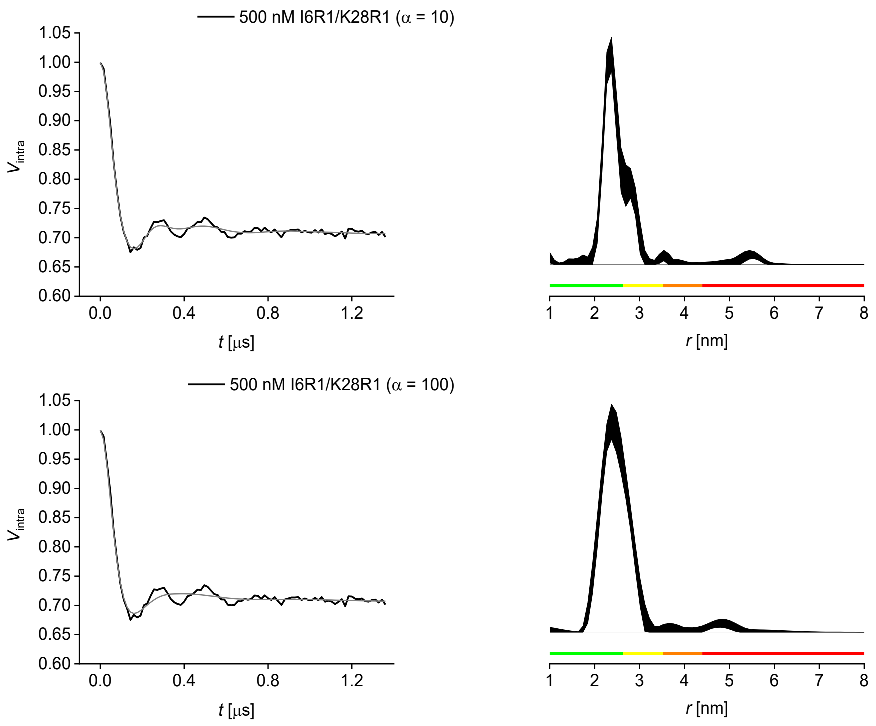

Figure S5: Comparison of PELDOR data obtained at 500 nM concentration using larger regularization parameters α. Top: α = 10; bottom: α = 100. Left: background-corrected data (black) with fit (grey); right: corresponding distance distributions given as 95% confidence intervals (± 2σ) with 50% noise added for error estimation during statistical analysis. Color bars represent reliability ranges (green: shape reliable; yellow: mean and width reliable; orange: mean reliable: red: no quantification possible). As expected, the larger α oversmooth the data and as a result the bimodality is lost, similar to the distribution observed for the 100 nM sample.

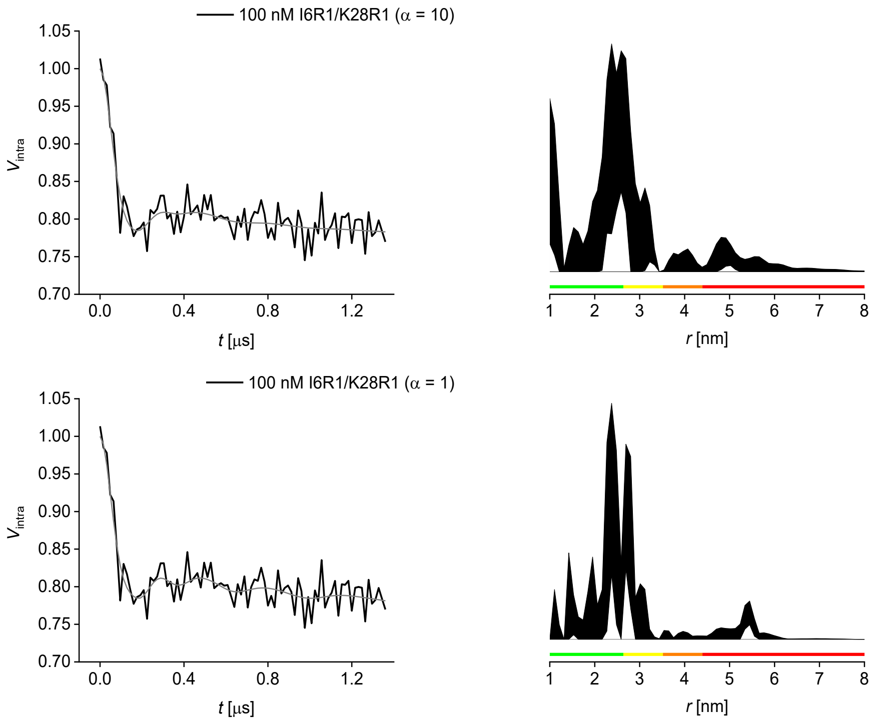

Figure S6: Comparison of PELDOR data obtained at 100 nM concentration using smaller regularization parameters α. Top: α = 10; bottom: α = 1. Left: background-corrected data (black) with fit (grey); right: corresponding distance distributions given as 95% confidence intervals (± 2σ) with 50% noise added for error estimation during statistical analysis. Color bars represent reliability ranges (green: shape reliable; yellow: mean and width reliable; orange: mean reliable: red: no quantification possible). With the smaller α some probability for bimodality is observed, however the SNR is too poor to be certain.

4. Deep neural network processing and wavelet denoising

100 and 500 nM PELDOR data (I6R1/K28R1 construct) and 500 nM RIDME data (I6H/N8H/K28H/Q32H construct) were further subjected to alternative processing methods.

Deep neural network processing was performed using DEERNet[2] within the Spinach[3] spin dynamics software (version 2.5.5459, Nov 2020). Input data were the same as used for the DeerAnalysis processing, but the time axis was supplied from 0 to t_{max} as required (i.e., the negative points of the time trace were removed).
DEERNet results of PELDOR data confirm DeerAnalysis results in that the second conformation is clearly distinguishable at 500 nM GB1 concentration, while at 100 nM the 95% confidence band would allow rejecting a second conformation (i.e., it is possible to draw a line without a second conformation present without leaving the confidence band).

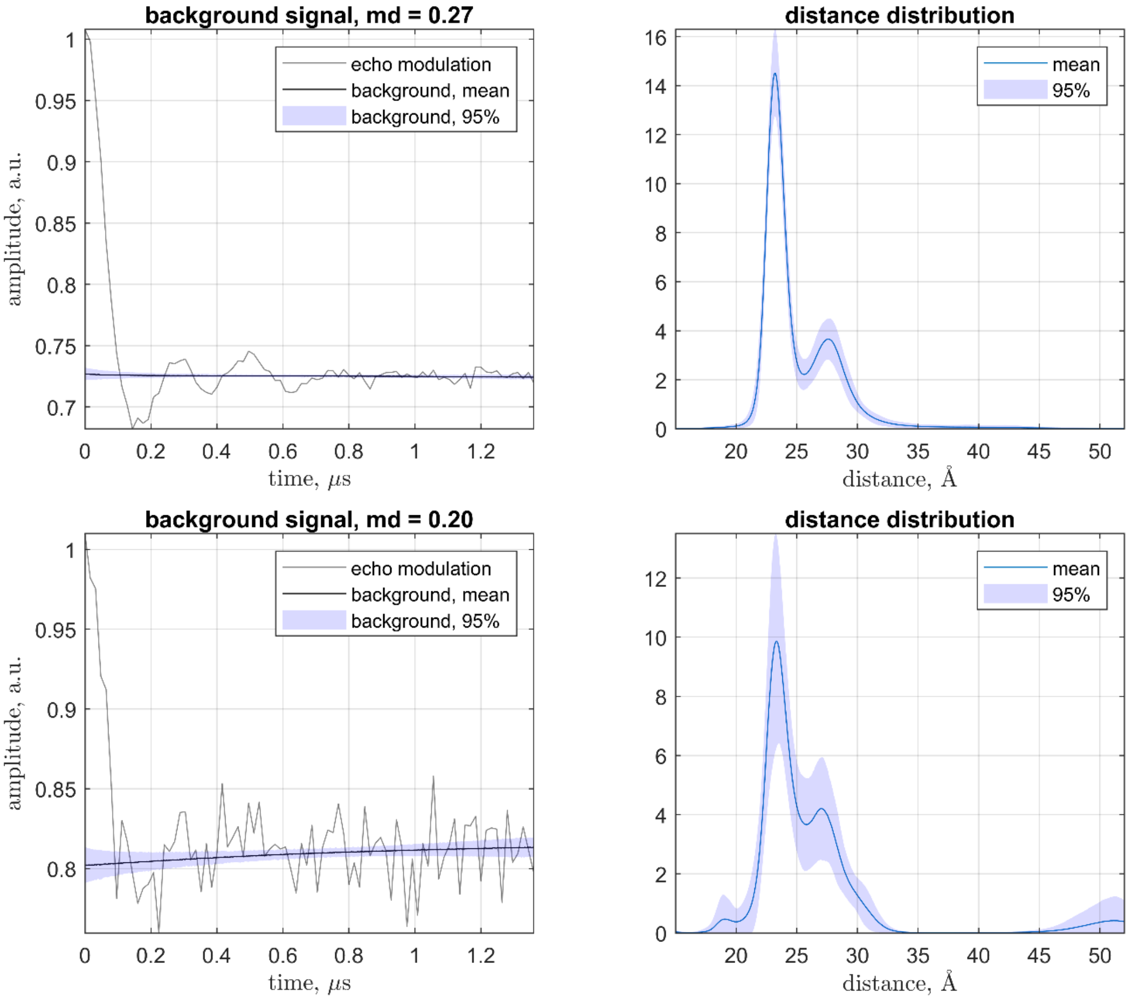

Figure S7: DEERNet results for 500 nM (top) and 100 nM (bottom) GB1 I6R1/K28R1 PELDOR data.

DEERNet results for the RIDME data show that the additional distance ~3 nm observed after DeerAnalysis processing is fully uncertain (and thus, potentially not real), confirming the RIDME data obtained for the 500 µM sample.

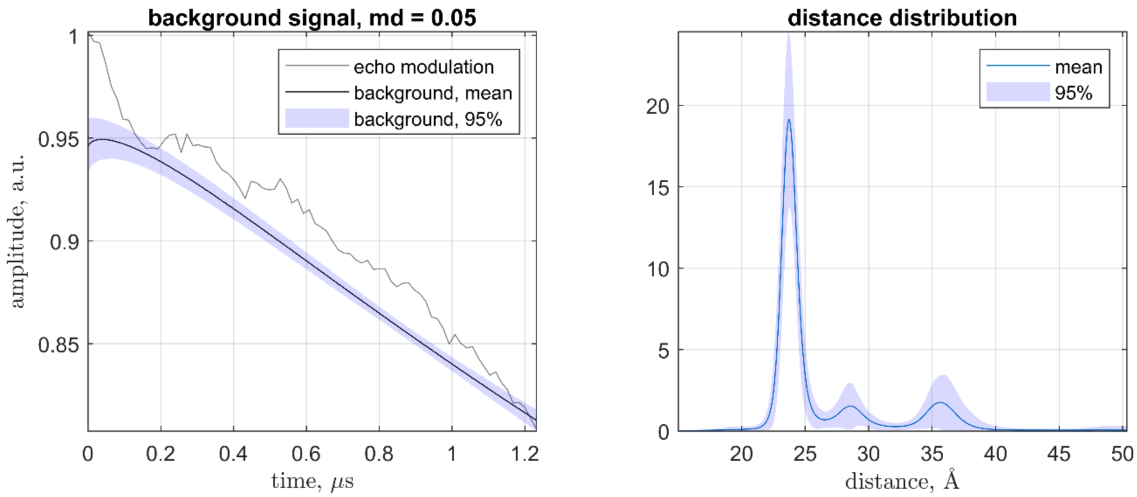

Figure S8: DEERNet results for 500 nM GB1 I6H/N8H/K28H/Q32H (1.6 μM Cu^{II}-NTA) RIDME data.

Wavelet denoising was performed using WavPDS (db6 wavelet).[4] Input data were the same as used for the DeerAnalysis processing, but the RIDME data were provided background-corrected (.fit file) to avoid background issues. After denoising, data were subjected to Tikhonov regularization within DeerAnalysis without further background correction.
As observed for the original PELDOR data, in our hands the bimodal distribution was not recovered at 100 nM protein concentration after denoising.

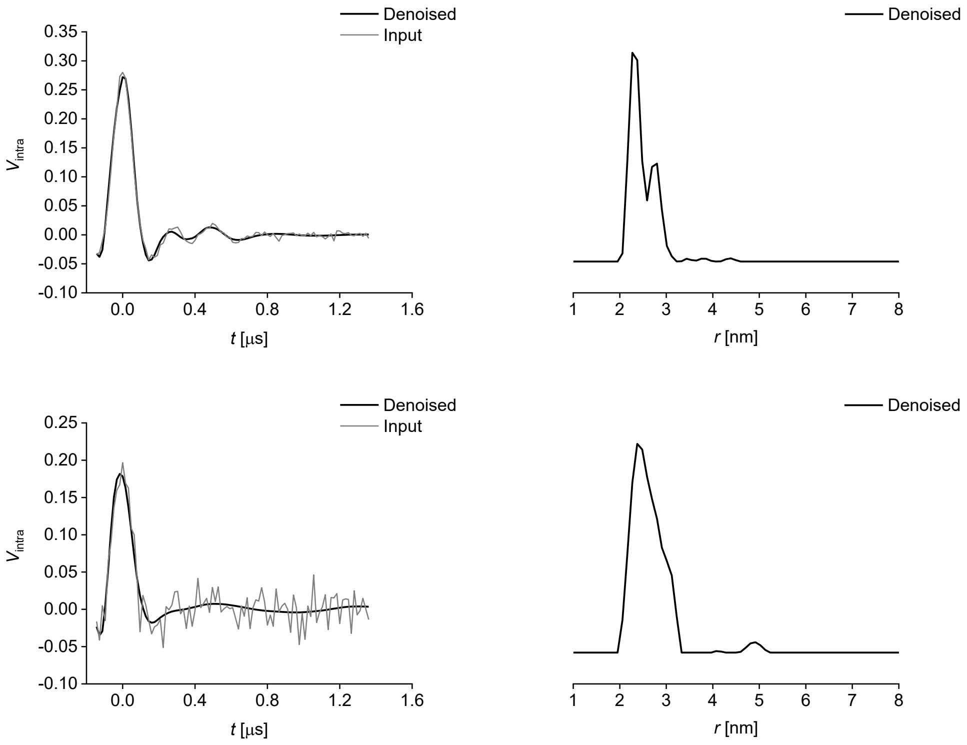

Figure S9: WavPDS results for 500 nM (top) and 100 nM (bottom) GB1 I6R1/K28R1 PELDOR data.

As observed with the original RIDME data, in our hands an artefact peak at ~3 nm is preserved after denoising.

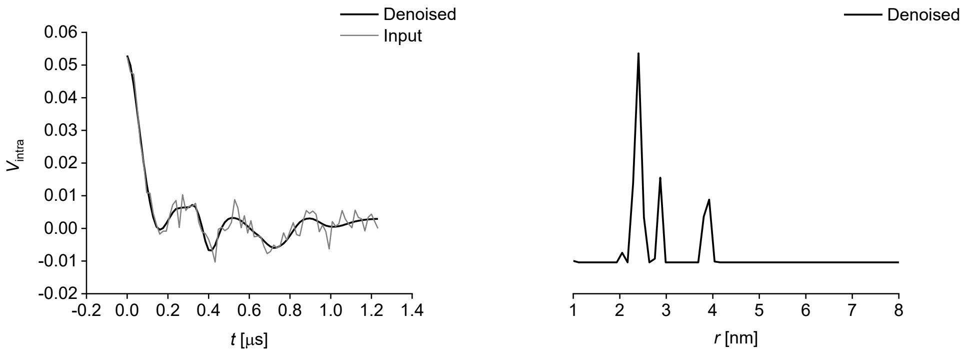

Figure S10: WavPDS results for 500 nM GB1 I6H/N8H/K28H/Q32H (1.6 μM Cu^{II}-NTA) RIDME data.

5. Instantaneous diffusion

The effect of instantaneous diffusion (ID;) on signal decay was determined as described previously varying the flip-angle of the second pulse of Hahn echo from π to π/5.[5] All measurements were recorded at the magnetic field where the signal maximum was found in the field swept EPR spectrum. No ID was observable at 500 nM protein concentration, so experiments were repeated at 25 μM protein concentration, however there was still no observable ID. This suggests that the effect of two spin labels in the same molecule is negligible in terms of dipolar dephasing in the system under study.

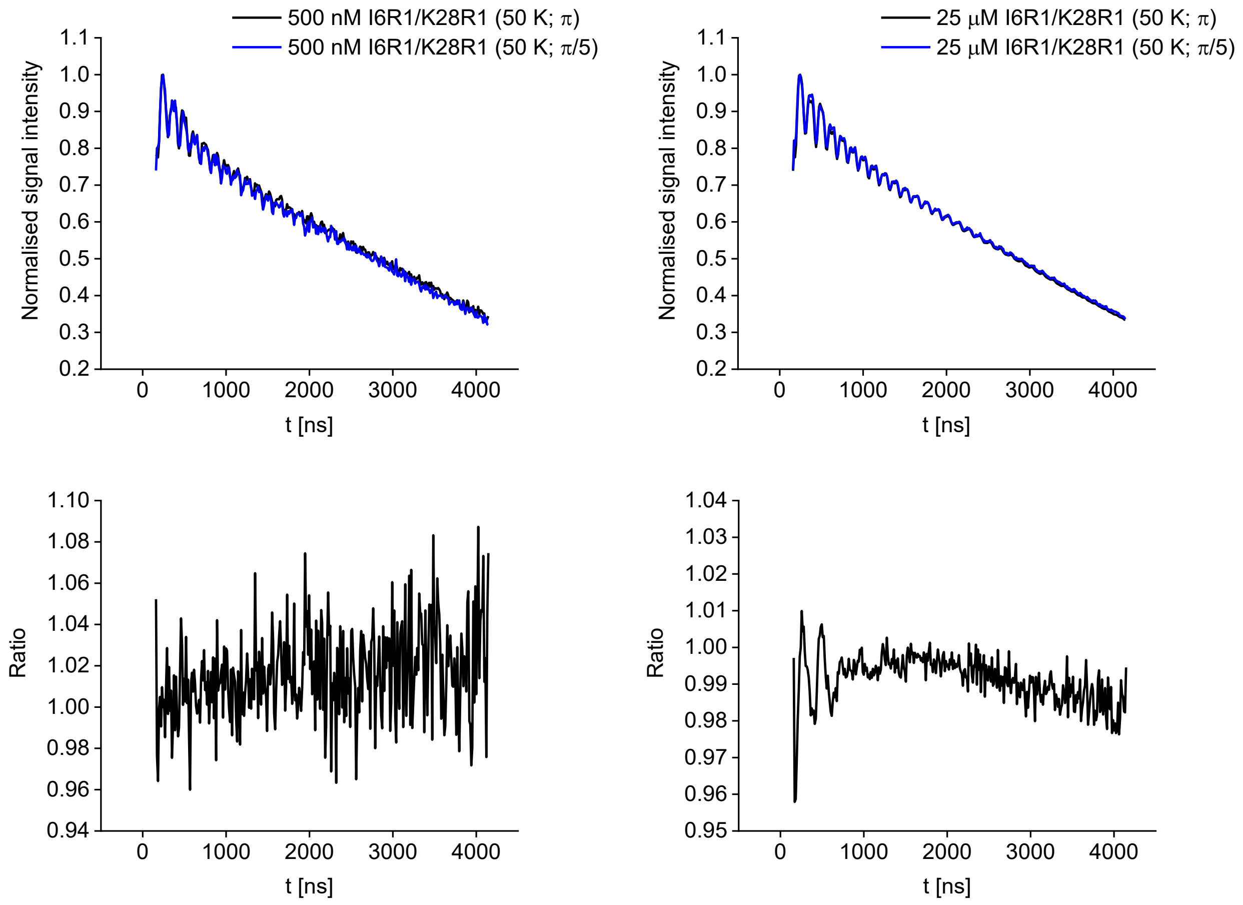

Figure S11: Decay traces from ID experiments on GB1 I6R1/K28R1 at 500 nM protein concentration (top left) and 25 μM protein concentration (top right), comparing π (black) and π/5 (blue) decay rates obtained at 50 K. Corresponding ratios are shown in the bottom row.

6. Sensitivity considerations

Our aim is to better understand why different experiments require different concentrations to achieve similar sensitivities. The relative performance of Cu^{II}-Cu^{II} PELDOR, Cu^{II}-Cu^{II} RIDME, and Cu^{II}-nitroxide RIDME has been quantified previously.[1] Here, we investigate how nitroxide-nitroxide PELDOR ranks in comparison. All sensitivity calculations were performed as outlined in previous reports.[1, 5]

"Dummy" PELDOR and RIDME experiments

Dummy PELDOR and RIDME traces were recorded for the I6R1/K28R1 construct at 500 nM and 25 μM protein concentration. The total number of echoes per point for each trace was kept constant (four shots per point in a 2-step phase cycle for PELDOR and one shot per point in an 8-step phase cycle for RIDME) and calculated noise levels (RMSD, root mean square deviation, estimated from the second and third quartile of the imaginary part of the phase-corrected trace) for each trace are compared.

At 500 nM protein concentration, results show the lowest noise for HQ (high Q, critically coupled resonator) RIDME while noise is approximately a factor 2.6 higher for LQ (low Q, over-coupled resonator) RIDME and a factor ~3.9 higher for PELDOR.

Experiment	RMSD estimate	Relative noise
PELDOR	0.11	3.89
RIDME, LQ	0.077	2.62
RIDME, HQ	0.029	1.00

Table S5: Estimated noise of dummy nitroxide-nitroxide PELDOR and RIDME experiments performed on the GB1 I6R1/K28R1 construct at 500 nM concentration.

Results suggest critical coupling gains a factor of ~2.6 in the single frequency experiment whereas off-resonance detection only loses a factor of ~1.5 (4-pulse DEER versus 5-pulse RIDME sequences over-coupled). This does not consider effects of modulation depth and signal averaging (shot repetition time) on sensitivities.

At 25 μM protein concentration, this is less clear-cut. Very low RMSD values ranging from 0.14 % to 0.38 % in a single scan lead to baseline imperfections contributing to the RMSD, which therefore does not reflect pure thermal noise anymore. The above factors change to 1.4 and 1.9, respectively, reproducing the same qualitative trend.

Experiment	RMSD estimate	Relative noise
PELDOR	0.0038	2.59
RIDME, LQ	0.0020	1.39
RIDME, HQ	0.0015	1.00

Table S6: Estimated noise of dummy nitroxide-nitroxide PELDOR and RIDME experiments performed on the GB1 I6R1/K28R1 construct at 25 μM concentration.

Sensitivity considerations for nitroxide-nitroxide PELDOR versus Cu^{II}-Cu^{II} RIDME experiments

Sensitivity (S) is determined as the ratio of modulation depth (Δ) divided by RMSD. Normalized sensitivity (S_n) is S divided by the square root of total echoes per point (taken as the product of number of scans, shots per point, number of τ averages, and phase cycle). Sensitivity per unit time (S_t) is S_n multiplied with the square root of the averaging rate.

Below, results are shown for comparison of nitroxide-nitroxide PELDOR at 100 nM and 500 nM protein concentration (GB1 I6R1/K28R1 construct) and Cu^{II}-Cu^{II} RIDME at 500 nM protein concentration (GB1 I6H/N8H/K28H/Q32H construct).

Experiment	Concentration	RMSD estimate	Δ	Averaging rate
NO-NO PELDOR	100 nM	0.021	0.223	200
NO-NO PELDOR	500 nM	0.0034	0.292	200
Cu^{II}-Cu^{II} RIDME	500 nM	0.0031	0.055	2500

Table S7: Noise estimates, modulation depths (Δ) and averaging rates for nitroxide-nitroxide PELDOR and Cu^{II}-Cu^{II} RIDME.

Experiment	Concentration	S	S_n	S_t	Relative S_t
NO-NO PELDOR	100 nM	10.43	0.02	0. 30	7
NO-NO PELDOR	500 nM	86.02	0.32	4.46	100
Cu^{II}-Cu^{II} RIDME	500 nM	17.58	0.009	0.45	10

Table S8: Sensitivities for nitroxide-nitroxide PELDOR and Cu^{II}-Cu^{II} RIDME.

Regarding the PELDOR measurements, one would expect the relative S_t of the 100 nM nitroxide-nitroxide PELDOR to be approximately 20, i.e., a factor 5 lower than at 500 nM. However, observed S_t at low concentration is another factor ~3 worse, which can be attributed to difficulties in optimization (pulses, phase, field position etc.) due to the very low signal; a slight shift in field position away from the maximum of the field swept spectrum would also explain the reduced modulation depth. This can therefore be considered a 'penalty' on achievable sensitivity at very low concentrations.

Regarding the Cu^{II}-Cu^{II} RIDME, the affinity of the Cu^{II}-NTA for the double-histidine site is currently limiting sensitivity, as indicated by a modulation depth of ~5% instead of ~25%. One solution would be either to covalently bind copper ions or to replace one double-histidine site with a nitroxide for Cu^{II}-nitroxide RIDME. In the latter case, a saturation of the double-histidine site is possible with similar sensitivity S_t compared to Cu^{II}-Cu^{II} RIDME. Thus, calculating with a modulation depth of 25%, the relative S_t would go up by a factor 5 from 10 to 50 at 500 nM, and thus would be ~10 at 100 nM compared to 7 for the corresponding nitroxide-nitroxide PELDOR. We therefore postulate that Cu^{II}-nitroxide RIDME measurements should be possible at protein concentrations of 100 nM (or less).

Simulated modulation depth profile for the tetra-histidine construct

The observed modulation depth of ~5.5% for the Cu^{II}-Cu^{II} RIDME measurement is consistent with prediction, based on the simulated sensitivity profile shown below.[6] The maximum of the profile is 0.22, which for a maximum theoretical modulation depth of 25.7% yields an expected modulation depth of 5.7%. Since the labelling efficiency is limited by the presence of the β-sheet double-histidine motif, simulation of a sensitivity profile for a tetra-histidine construct containing two α-helical double-histidine motifs was performed. The predicted maximum of the profile is ~0.50, which for a maximum theoretical modulation depth of 25.7% yields an expected modulation depth of >12%.

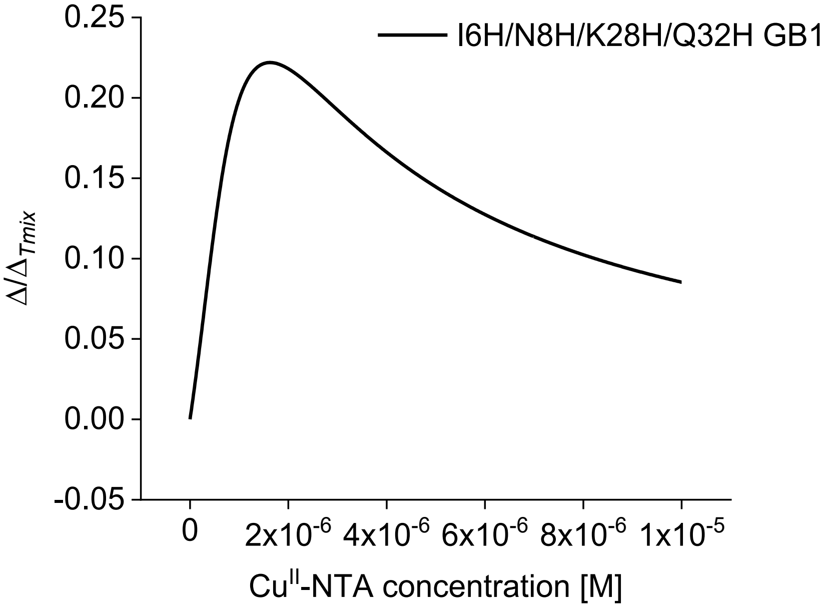

Figure S12: 500 nM tetra-histidine GB1 simulated modulation depth profile. The simulated modulation depth profile is shown as a function of increasing Cu^{II}-NTA concentration. Dissociation constants (K_D) of 0.14 and 1.4 µM are assumed for the α-helical and β-sheet double-histidine motifs, respectively.

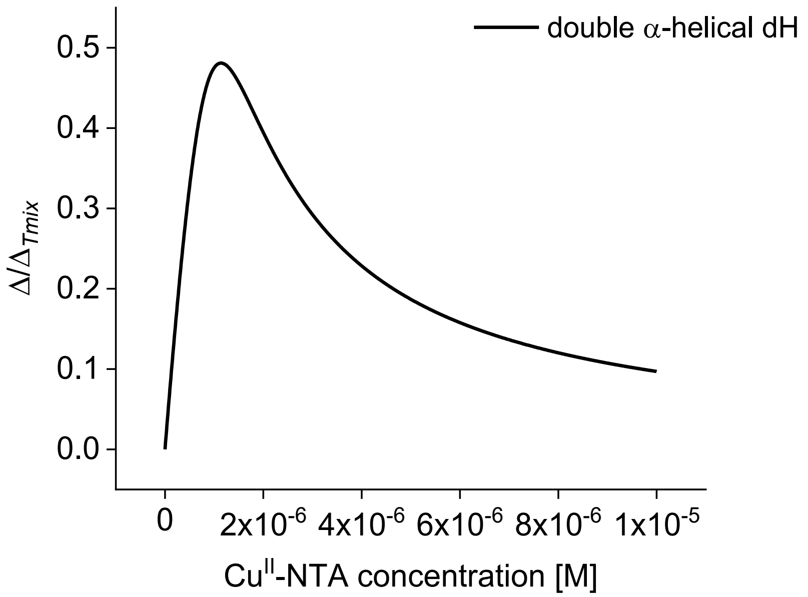

Figure S13: Simulated modulation depth profile of a double α-helical double-histidine construct, at 500 nM protein concentration. The simulated modulation depth profile is shown as a function of increasing Cu^{II}-NTA concentration. A dissociation constant (K_D) of 0.14 µM is assumed for each of the α-helical double-histidine motifs.

7. Literature search

Nitroxide-nitroxide PELDOR

PubMed was searched on 30 November 2020 for the search terms “PELDOR” or “DEER” and “EPR”. The time range was 2017 to 2020. The search resulted in 124 publications, including 9 reviews, which were not further investigated. 61 out of the remaining 115 publications showing nitroxide-nitroxide PELDOR data on biomolecules (protein, DNA, or RNA) were used for statistical analysis.[5, 7-66]

All these 61 publications provided details regarding biomolecule and spin concentration, and we had excluded any spin concentrations above 400 micromolar to not artificially inflate the average.

We found an average (mean) spin concentration of 116 μM, a minimum of 5 μM in one case, and as stated above the maximum was set by us to 400 μM. The standard deviation was 90 μM, showing the large spread across values, with the median being at 100 μM spin concentration.

Metal-metal RIDME

On 22 January 2021, the Milikisyants paper[67] introducing the 5-pulse RIDME sequence had 91 citations. Out of these citations, 13 papers described metal-metal RIDME data and provided details regarding bi-omolecule or chemical compound and spin concentration and were used for statistical analysis.[1, 68-79]

We found an average (mean) spin concentration of 253 μM, a minimum of 25 μM in one case, and a maximum of 1000 μM. The standard deviation was 259 μM, showing the large spread across values, with the median being at 100 μM spin concentration.

8. References

(1) Wort, J. L.; Ackermann, K.; Giannoulis, A.; Stewart, A. J.; Norman, D. G.; Bode, B. E. Sub-Micromolar Pulse Dipolar EPR Spectroscopy Reveals Increasing Cu^{II}-labelling of Double-Histidine Motifs with Lower Temperature. *Angew. Chem. Int. Ed.* **2019,** *58*, 11681-11685 https://doi.org/10.1002/anie.201904848.

(2) Worswick, S. G.; Spencer, J. A.; Jeschke, G.; Kuprov, I. Deep Neural Network Processing of DEER Data. *Sci. Adv.* **2018,** *4*, eaat5218 https://doi.org/10.1126/sciadv.aat5218.

(3) Hogben, H. J.; Krzystyniak, M.; Charnock, G. T.; Hore, P. J.; Kuprov, I. Spinach--a Software Library for Simulation of Spin Dynamics in Large Spin Systems. *J. Magn. Reson.* **2011,** *208*, 179-94 https://doi.org/10.1016/j.jmr.2010.11.008.

(4) Srivastava, M.; Georgieva, E. R.; Freed, J. H. A New Wavelet Denoising Method for Experimental Time-Domain Signals: Pulsed Dipolar Electron Spin Resonance. *J. Phys. Chem. A* **2017,** *121*, 2452-2465 https://doi.org/10.1021/acs.jpca.7b00183.

(5) Ackermann, K.; Pliotas, C.; Valera, S.; Naismith, J. H.; Bode, B. E. Sparse Labeling PELDOR Spectroscopy on Multimeric Mechanosensitive Membrane Channels. *Biophys. J.* **2017,** *113*, 1968-1978 https://doi.org/10.1016/j.bpj.2017.09.005.

(6) Wort, J. L.; Ackermann, K.; Norman, D. G.; Bode, B. E. A General Model to Optimise Cu^{II} Labelling Efficiency of Double-Histidine Motifs for Pulse Dipolar EPR Applications. *Phys. Chem. Chem. Phys.* **2021,** *23*, 3810-3819 https://doi.org/10.1039/D0CP06196D.

(7) Abdullin, D.; Fleck, N.; Klein, C.; Brehm, P.; Spicher, S.; Lützen, A.; Grimme, S.; Schiemann, O. Synthesis of μ2-Oxo-Bridged Iron(III) Tetraphenylporphyrin–Spacer–Nitroxide Dimers and their Structural and Dynamics Characterization by using EPR and MD Simulations. *Chem. Eur. J.* **2019,** *25*, 2586-2596 https://doi.org/10.1002/chem.201805016.

(8) Assafa, T. E.; Anders, K.; Linne, U.; Essen, L.-O.; Bordignon, E. Light-Driven Domain Mechanics of a Minimal Phytochrome Photosensory Module Studied by EPR. *Structure* **2018,** *26*, 1534-1545.e4 https://doi.org/10.1016/j.str.2018.08.003.

(9) Assafa, T. E.; Nandi, S.; Śmiłowicz, D.; Galazzo, L.; Teucher, M.; Elsner, C.; Pütz, S.; Bleicken, S.; Robin, A. Y.; Westphal, D. et al. Biophysical Characterization of Pro-apoptotic BimBH3 Peptides Reveals an Unexpected Capacity for Self-Association. *Structure* **2020**, https://doi.org/10.1016/j.str.2020.09.002.

(10) Barnes, C. A.; Mishra, P.; Baber, J. L.; Strub, M.-P.; Tjandra, N. Conformational Heterogeneity in the Activation Mechanism of Bax. *Structure* **2017,** *25*, 1310-1316.e3 https://doi.org/10.1016/j.str.2017.06.009.

(11) Braun, T. S.; Widder, P.; Osswald, U.; Groß, L.; Williams, L.; Schmidt, M.; Helmle, I.; Summerer, D.; Drescher, M. Isoindoline-Based Nitroxides as Bioresistant Spin Labels for Protein Labeling through Cysteines and Alkyne-Bearing Noncanonical Amino Acids. *ChemBioChem* **2020,** *21*, 958-962 https://doi.org/10.1002/cbic.201900537.

(12) Carey Hulyer, A. R.; Briggs, D. A.; O'Mara, M. L.; Kerr, I. D.; Harmer, J. R.; Callaghan, R. Cross-Linking, DEER-Spectroscopy and Molecular Dynamics Confirm the Inward Facing State of P-Glycoprotein in a Lipid Membrane. *J. Struct. Biol.* **2020,** *211*, 107513 https://doi.org/10.1016/j.jsb.2020.107513.

(13) Chiliveri, S. C.; Louis, J. M.; Ghirlando, R.; Baber, J. L.; Bax, A. Tilted, Uninterrupted, Monomeric HIV-1 gp41 Transmembrane Helix from Residual Dipolar Couplings. *J. Am. Chem. Soc.* **2018,** *140*, 34-37 https://doi.org/10.1021/jacs.7b10245.

(14) Collauto, A.; von Bülow, S.; Gophane, D. B.; Saha, S.; Stelzl, L. S.; Hummer, G.; Sigurdsson, S. T.; Prisner, T. F. Compaction of RNA Duplexes in the Cell. *Angew. Chem. Int. Ed.* **2020,** *59*, 23025-23029 https://doi.org/10.1002/anie.202009800.

(15) Domnick, C.; Eggert, F.; Wuebben, C.; Bornewasser, L.; Hagelueken, G.; Schiemann, O.; Kath-Schorr, S. EPR Distance Measurements on Long Non-coding RNAs Empowered by Genetic Alphabet Expansion Transcription. *Angew. Chem. Int. Ed.* **2020,** *59*, 7891-7896 https://doi.org/10.1002/anie.201916447.

(16) Domnick, C.; Hagelueken, G.; Eggert, F.; Schiemann, O.; Kath-Schorr, S. Posttranscriptional Spin Labeling of RNA by Tetrazine-Based Cycloaddition. *Org. Biomol. Chem.* **2019,** *17*, 1805-1808 https://doi.org/10.1039/C8OB02597E.

(17) Erlenbach, N.; Grünewald, C.; Krstic, B.; Heckel, A.; Prisner, T. F. 'End-to-End' Stacking of Small dsRNA. *RNA* **2018**, https://doi.org/10.1261/rna.068130.118.

(18) Galazzo, L.; Maso, L.; De Rosa, E.; Bortolus, M.; Doni, D.; Acquasaliente, L.; De Filippis, V.; Costantini, P.; Carbonera, D. Identifying Conformational Changes with Site-Directed Spin Labeling Reveals that the GTPase Domain of HydF is a Molecular Switch. *Sci. Rep.* **2017,** *7*, 1714 https://doi.org/10.1038/s41598-017-01886-y.

(19) Galazzo, L.; Meier, G.; Timachi, M. H.; Hutter, C. A. J.; Seeger, M. A.; Bordignon, E. Spin-Labeled Nanobodies as Protein Conformational Reporters for Electron Paramagnetic Resonance in Cellular Membranes. *Proc. Natl. Acad. Sci. USA* **2020,** *117*, 2441-2448 https://doi.org/10.1073/pnas.1913737117.

(20) Giannoulis, A.; Ackermann, K.; Spindler, P. E.; Higgins, C.; Cordes, D. B.; Slawin, A. M. Z.; Prisner, T. F.; Bode, B. E. Nitroxide–Nitroxide and Nitroxide–Metal Distance Measurements in Transition Metal Complexes with Two or Three Paramagnetic Centres Give Access to Thermodynamic and Kinetic Stabilities. *Phys. Chem. Chem. Phys.* **2018,** *20*, 11196-11205 https://doi.org/10.1039/C8CP01611A.

(21) Giannoulis, A.; Feintuch, A.; Barak, Y.; Mazal, H.; Albeck, S.; Unger, T.; Yang, F.; Su, X.-C.; Goldfarb, D. Two Closed ATP- and ADP-Dependent Conformations in Yeast Hsp90 Chaperone Detected by Mn(II) EPR Spectroscopic Techniques. *Proc. Natl. Acad. Sci. USA* **2020,** *117*, 395-404 https://doi.org/10.1073/pnas.1916030116.

(22) Gmeiner, C.; Dorn, G.; Allain, F. H. T.; Jeschke, G.; Yulikov, M. Spin Labelling for Integrative Structure Modelling: a Case Study of the Polypyrimidine-Tract Binding Protein 1 Domains in Complexes with Short RNAs. *Phys. Chem. Chem. Phys.* **2017,** *19*, 28360-28380 https://doi.org/10.1039/C7CP05822E.

(23) Gmeiner, C.; Klose, D.; Mileo, E.; Belle, V.; Marque, S. R. A.; Dorn, G.; Allain, F. H. T.; Guigliarelli, B.; Jeschke, G.; Yulikov, M. Orthogonal Tyrosine and Cysteine Site-Directed Spin Labeling for Dipolar Pulse EPR Spectroscopy on Proteins. *J. Phys. Chem. Lett.* **2017,** *8*, 4852-4857 https://doi.org/10.1021/acs.jpclett.7b02220.

(24) Gophane, D. B.; Endeward, B.; Prisner, T. F.; Sigurdsson, S. T. A Semi-Rigid Isoindoline-Derived Nitroxide Spin Label for RNA. *Org. Biomol. Chem.* **2018,** *16*, 816-824 https://doi.org/10.1039/C7OB02870A.

(25) Gränz, M.; Erlenbach, N.; Spindler, P.; Gophane, D. B.; Stelzl, L. S.; Sigurdsson, S. T.; Prisner, T. F. Dynamics of Nucleic Acids at Room Temperature Revealed by Pulsed EPR Spectroscopy. *Angew. Chem. Int. Ed.* **2018,** *57*, 10540-10543 https://doi.org/10.1002/anie.201803682.

(26) Gruian, C. M.; Rickert, C.; Nicklisch, S. C. T.; Vanea, E.; Steinhoff, H.-J.; Simon, S. Conformational Changes and Competitive Adsorption between Serum Albumin and Hemoglobin on Bioceramic Substrates. *ChemPhysChem* **2017,** *18*, 634-642 https://doi.org/10.1002/cphc.201600886.

(27) Grytz, C. M.; Kazemi, S.; Marko, A.; Cekan, P.; Güntert, P.; Sigurdsson, S. T.; Prisner, T. F. Determination of Helix Orientations in a Flexible DNA by Multi-Frequency EPR Spectroscopy. *Phys. Chem. Chem. Phys.* **2017,** *19*, 29801-29811 https://doi.org/10.1039/C7CP04997H.

(28) Heinz, M.; Erlenbach, N.; Stelzl, L. S.; Thierolf, G.; Kamble, N. R.; Sigurdsson, S. T.; Prisner, T. F.; Hummer, G. High-Resolution EPR Distance Measurements on RNA and DNA with the Non-Covalent Ǵ Spin Label. *Nucleic Acids Res.* **2019,** *48*, 924-933 https://doi.org/10.1093/nar/gkz1096.
(29) Jassoy, J. J.; Heubach, C. A.; Hett, T.; Bernhard, F.; Haege, F. R.; Hagelueken, G.; Schiemann, O. Site Selective and Efficient Spin Labeling of Proteins with a Maleimide-Functionalized Trityl Radical for Pulsed Dipolar EPR Spectroscopy. *Molecules* **2019,** *24*, 2735 https://doi.org/10.3390/molecules24152735.
(30) Jaumann, E. A.; Steinwand, S.; Klenik, S.; Plackmeyer, J.; Bats, J. W.; Wachtveitl, J.; Prisner, T. F. A Combined Optical and EPR Spectroscopy Study: Azobenzene-Based Biradicals as Reversible Molecular Photoswitches. *Phys. Chem. Chem. Phys.* **2017,** *19*, 17263-17269 https://doi.org/10.1039/C7CP02088K.
(31) Kacprzak, S.; Njimona, I.; Renz, A.; Feng, J.; Reijerse, E.; Lubitz, W.; Krauss, N.; Scheerer, P.; Nagano, S.; Lamparter, T. et al. Intersubunit Distances in Full-Length, Dimeric, Bacterial Phytochrome Agp1, as Measured by Pulsed Electron-Electron Double Resonance (PELDOR) between Different Spin Label Positions, Remain Unchanged upon Photoconversion. *J. Biol. Chem.* **2017,** *292*, 7598-7606 https://doi.org/10.1074/jbc.M116.761882.
(32) Kumar, P.; van Son, M.; Zheng, T.; Valdink, D.; Raap, J.; Kros, A.; Huber, M. Coiled-Coil Formation of the Membrane-Fusion K/E Peptides Viewed by Electron Paramagnetic Resonance. *PLoS One* **2018,** *13*, e0191197 https://doi.org/10.1371/journal.pone.0191197.
(33) Laugwitz, J. M.; Haeri, H. H.; Kaiser, A.; Krug, U.; Hinderberger, D.; Beck-Sickinger, A. G.; Schmidt, P. Probing the Y2 Receptor on Transmembrane, Intra- and Extra-Cellular Sites for EPR Measurements. *Molecules* **2020,** *25*, 4143 https://doi.org/10.3390/molecules25184143.
(34) Levy, A. R.; Turgeman, M.; Gevorkyan-Aiapetov, L.; Ruthstein, S. The Structural Flexibility of the Human Copper Chaperone Atox1: Insights from Combined Pulsed EPR Studies and Computations. *Protein Sci.* **2017,** *26*, 1609-1618 https://doi.org/10.1002/pro.3197.
(35) Lim, S.; Roseman, G.; Peshenko, I.; Manchala, G.; Cudia, D.; Dizhoor, A. M.; Millhauser, G.; Ames, J. B. Retinal Guanylyl Cyclase Activating Protein 1 Forms a Functional Dimer. *PLoS One* **2018,** *13*, e0193947 https://doi.org/10.1371/journal.pone.0193947.
(36) Malygin, A. A.; Krumkacheva, O. A.; Graifer, D. M.; Timofeev, I. O.; Ochkasova, A. S.; Meschaninova, M. I.; Venyaminova, A. G.; Fedin, M. V.; Bowman, M.; Karpova, G. G. et al. Exploring the Interactions of Short RNAs with the Human 40S Ribosomal Subunit near the mRNA Entry Site by EPR Spectroscopy. *Nucleic Acids Res.* **2019,** *47*, 11850-11860 https://doi.org/10.1093/nar/gkz1039.
(37) Motion, C. L.; Cassidy, S. L.; Cruickshank, P. A. S.; Hunter, R. I.; Bolton, D. R.; El Mkami, H.; Van Doorslaer, S.; Lovett, J. E.; Smith, G. M. The use of Composite Pulses for Improving DEER Signal at 94GHz. *J. Magn. Reson.* **2017,** *278*, 122-133 https://doi.org/10.1016/j.jmr.2017.03.018.
(38) Pliotas, C. Ion Channel Conformation and Oligomerization Assessment by Site-Directed Spin Labeling and Pulsed-EPR. *Methods Enzymol.* **2017,** *594*, 203-242 https://doi.org/10.1016/bs.mie.2017.05.013.
(39) Pribitzer, S.; Sajid, M.; Hülsmann, M.; Godt, A.; Jeschke, G. Pulsed Triple Electron Resonance (TRIER) for Dipolar Correlation Spectroscopy. *J. Magn. Reson.* **2017,** *282*, 119-128 https://doi.org/10.1016/j.jmr.2017.07.012.
(40) Reichenwallner, J.; Thomas, A.; Steinbach, T.; Eisermann, J.; Schmelzer, C. E. H.; Wurm, F.; Hinderberger, D. Ligand-Binding Cooperativity Effects in Polymer–Protein Conjugation. *Biomacromolecules* **2019,** *20*, 1118-1131 https://doi.org/10.1021/acs.biomac.9b00016.
(41) Saha, S.; Hetzke, T.; Prisner, T. F.; Sigurdsson, S. T. Noncovalent Spin-Labeling of RNA: The Aptamer Approach. *Chem. Commun.* **2018,** *54*, 11749-11752 https://doi.org/10.1039/C8CC05597A.

(42) Sahu, I. D.; Dixit, G.; Reynolds, W. D.; Kaplevatsky, R.; Harding, B. D.; Jaycox, C. K.; McCarrick, R. M.; Lorigan, G. A. Characterization of the Human KCNQ1 Voltage Sensing Domain (VSD) in Lipodisq Nanoparticles for Electron Paramagnetic Resonance (EPR) Spectroscopic Studies of Membrane Proteins. *J. Phys. Chem. B* **2020,** *124*, 2331-2342 https://doi.org/10.1021/acs.jpcb.9b11506.

(43) Sameach, H.; Narunsky, A.; Azoulay-Ginsburg, S.; Gevorkyan-Aiapetov, L.; Zehavi, Y.; Moskovitz, Y.; Juven-Gershon, T.; Ben-Tal, N.; Ruthstein, S. Structural and Dynamics Characterization of the MerR Family Metalloregulator CueR in its Repression and Activation States. *Structure* **2017,** *25*, 988-996.e3 https://doi.org/10.1016/j.str.2017.05.004.

(44) Schmidt, T.; Clore, G. M. T_m Filtering by ^{1}H-Methyl Labeling in a Deuterated Protein for Pulsed Double Electron–Electron Resonance EPR. *Chem. Commun.* **2020,** *56*, 10890-10893 https://doi.org/10.1039/D0CC04369A.

(45) Schmidt, T.; Jeon, J.; Okuno, Y.; Chiliveri, S. C.; Clore, G. M. Submillisecond Freezing Permits Cryoprotectant-Free EPR Double Electron−Electron Resonance Spectroscopy. *ChemPhysChem* **2020,** *21*, 1224-1229 https://doi.org/10.1002/cphc.202000312.

(46) Schmidt, T.; Louis, J. M.; Clore, G. M. Probing the Interaction between HIV-1 Protease and the Homodimeric p66/p66' Reverse Transcriptase Precursor by Double Electron-Electron Resonance EPR Spectroscopy. *ChemBioChem* **2020,** *21*, 3051-3055 https://doi.org/10.1002/cbic.202000263.

(47) Schmidt, T.; Schwieters, C. D.; Clore, G. M. Spatial Domain Organization in the HIV-1 Reverse Transcriptase p66 Homodimer Precursor Probed by Double Electron-Electron Resonance EPR. *Proc. Natl. Acad. Sci. USA* **2019,** *116*, 17809-17816 https://doi.org/10.1073/pnas.1911086116.

(48) Schultz, K. M.; Fischer, M. A.; Noey, E. L.; Klug, C. S. Disruption of the *E. coli* LptC Dimerization Interface and Characterization of Lipopolysaccharide and LptA Binding to Monomeric LptC. *Protein Sci.* **2018,** *27*, 1407-1417 https://doi.org/10.1002/pro.3429.

(49) Selmke, B.; Borbat, P. P.; Nickolaus, C.; Varadarajan, R.; Freed, J. H.; Trommer, W. E. Open and Closed Form of Maltose Binding Protein in Its Native and Molten Globule State As Studied by Electron Paramagnetic Resonance Spectroscopy. *Biochemistry* **2018,** *57*, 5507-5512 https://doi.org/10.1021/acs.biochem.8b00322.

(50) Shah, A.; Roux, A.; Starck, M.; Mosely, J. A.; Stevens, M.; Norman, D. G.; Hunter, R. I.; El Mkami, H.; Smith, G. M.; Parker, D. et al. A Gadolinium Spin Label with Both a Narrow Central Transition and Short Tether for Use in Double Electron Electron Resonance Distance Measurements. *Inorg. Chem.* **2019,** *58*, 3015-3025 https://doi.org/10.1021/acs.inorgchem.8b02892.

(51) Stevens, M.; Franke, B.; Skorupka, K. A.; Cafiso, D. S.; Pornillos, O.; Mayans, O.; Norman, D. G. Exploration of the TRIM Fold of MuRF1 Using EPR Reveals a Canonical Antiparallel Structure and Extended COS-Box. *J. Mol. Biol.* **2019,** *431*, 2900-2909 https://doi.org/10.1016/j.jmb.2019.05.025.

(52) Suliman, M.; Santosh, V.; Seegar, T. C. M.; Dalton, A. C.; Schultz, K. M.; Klug, C. S.; Barton, W. A. Directed Evolution Provides Insight into Conformational Substrate Sampling by SrtA. *PLoS One* **2017,** *12*, e0184271 https://doi.org/10.1371/journal.pone.0184271.

(53) Tessmer, M. H.; DeCero, S. A.; del Alamo, D.; Riegert, M. O.; Meiler, J.; Frank, D. W.; Feix, J. B. Characterization of the ExoU Activation Mechanism Using EPR and Integrative Modeling. *Sci. Rep.* **2020,** *10*, 19700 https://doi.org/10.1038/s41598-020-76023-3.

(54) Teucher, M.; Bordignon, E. Improved Signal Fidelity in 4-Pulse DEER with Gaussian Pulses. *J. Magn. Reson.* **2018,** *296*, 103-111 https://doi.org/10.1016/j.jmr.2018.09.003.

(55) Teucher, M.; Zhang, H.; Bader, V.; Winklhofer, K. F.; García-Sáez, A. J.; Rajca, A.; Bleicken, S.; Bordignon, E. A New Perspective on Membrane-Embedded Bax Oligomers Using DEER and Bioresistant Orthogonal Spin Labels. *Sci. Rep.* **2019,** *9*, 13013 https://doi.org/10.1038/s41598-019-49370-z.

(56) Tran, T. T.; Liu, Z.; Fanucci, G. E. Conformational Landscape of non-B Variants of HIV-1 Protease: A Pulsed EPR Study. *Biochem. Biophys. Res. Commun.* **2020,** *532*, 219-224 https://doi.org/10.1016/j.bbrc.2020.08.030.
(57) Velisetty, P.; Stein, R. A.; Sierra-Valdez, F. J.; Vásquez, V.; Cordero-Morales, J. F. Expression and Purification of the Pain Receptor TRPV1 for Spectroscopic Analysis. *Sci. Rep.* **2017,** *7*, 9861 https://doi.org/10.1038/s41598-017-10426-7.
(58) Wegner, J.; Valora, G.; Halbmair, K.; Kehl, A.; Worbs, B.; Bennati, M.; Diederichsen, U. Semi-Rigid Nitroxide Spin Label for Long-Range EPR Distance Measurements of Lipid Bilayer Embedded β-Peptides. *Chem. Eur. J.* **2019,** *25*, 2203-2207 https://doi.org/10.1002/chem.201805880.
(59) Weinrich, T.; Jaumann, E. A.; Scheffer, U.; Prisner, T. F.; Göbel, M. W. A Cytidine Phosphoramidite with Protected Nitroxide Spin Label: Synthesis of a Full-Length TAR RNA and Investigation by In-Line Probing and EPR Spectroscopy. *Chem. Eur. J.* **2018,** *24*, 6202-6207 https://doi.org/10.1002/chem.201800167.
(60) Weinrich, T.; Jaumann, E. A.; Scheffer, U. M.; Prisner, T. F.; Göbel, M. W. Phosphoramidite Building Blocks with Protected Nitroxides for the Synthesis of Spin-Labeled DNA and RNA. *Beilstein J. Org. Chem.* **2018,** *14*, 1563-1569 https://doi.org/10.3762/bjoc.14.133.
(61) Widder, P.; Schuck, J.; Summerer, D.; Drescher, M. Combining Site-Directed Spin Labeling *In Vivo* and In-Cell EPR Distance Determination. *Phys. Chem. Chem. Phys.* **2020,** *22*, 4875-4879 https://doi.org/10.1039/C9CP05584C.
(62) Wuebben, C.; Blume, S.; Abdullin, D.; Brajtenbach, D.; Haege, F.; Kath-Schorr, S.; Schiemann, O. Site-Directed Spin Labeling of RNA with a Gem-Diethylisoindoline Spin Label: PELDOR, Relaxation, and Reduction Stability. *Molecules* **2019,** *24*, 4482 https://doi.org/10.3390/molecules24244482.
(63) Yardeni, E. H.; Bahrenberg, T.; Stein, R. A.; Mishra, S.; Zomot, E.; Graham, B.; Tuck, K. L.; Huber, T.; Bibi, E.; McHaourab, H. S. et al. Probing the Solution Structure of the *E. coli* Multidrug Transporter MdfA Using DEER Distance Measurements with Nitroxide and Gd(III) Spin Labels. *Sci. Rep.* **2019,** *9*, 12528 https://doi.org/10.1038/s41598-019-48694-0.
(64) Ye, L.; Van Eps, N.; Li, X.; Ernst, O. P.; Prosser, R. S. Utilizing Tagged Paramagnetic Shift Reagents to Monitor Protein Dynamics by NMR. *Biochim. Biophys. Acta, Proteins Proteomics* **2017,** *1865*, 1555-1563 https://doi.org/10.1016/j.bbapap.2017.09.011.
(65) Yin, D. M.; Hannam, J. S.; Schmitz, A.; Schiemann, O.; Hagelueken, G.; Famulok, M. Studying the Conformation of a Receptor Tyrosine Kinase in Solution by Inhibitor-Based Spin Labeling. *Angew. Chem. Int. Ed.* **2017,** *56*, 8417-8421 https://doi.org/10.1002/anie.201703154.
(66) Zaccak, M.; Qasem, Z.; Gevorkyan-Airapetov, L.; Ruthstein, S. An EPR Study on the Interaction between the Cu(I) Metal Binding Domains of ATP7B and the Atox1 Metallochaperone. *Int. J. Mol. Sci.* **2020,** *21*, 5536 https://doi.org/10.3390/ijms21155536.
(67) Milikisyants, S.; Scarpelli, F.; Finiguerra, M. G.; Ubbink, M.; Huber, M. A Pulsed EPR Method to Determine Distances Between Paramagnetic Centers with Strong Spectral Anisotropy and Radicals: The Dead-time Free RIDME Sequence. *J. Magn. Reson.* **2009,** *201*, 48-56 https://doi.org/10.1016/j.jmr.2009.08.008.
(68) Akhmetzyanov, D.; Ching, H. Y.; Denysenkov, V.; Demay-Drouhard, P.; Bertrand, H. C.; Tabares, L. C.; Policar, C.; Prisner, T. F.; Un, S. RIDME Spectroscopy on High-Spin Mn^{2+} Centers. *Phys. Chem. Chem. Phys.* **2016,** *18*, 30857-30866 https://doi.org/10.1039/c6cp05239h.
(69) Azarkh, M.; Bieber, A.; Qi, M.; Fischer, J. W. A.; Yulikov, M.; Godt, A.; Drescher, M. Gd(III)-Gd(III) Relaxation-Induced Dipolar Modulation Enhancement for In-Cell Electron Paramagnetic Resonance Distance Determination. *J. Phys. Chem. Lett.* **2019,** *10*, 1477-1481 https://doi.org/10.1021/acs.jpclett.9b00340.

(70) Breitgoff, F. D.; Keller, K.; Qi, M.; Klose, D.; Yulikov, M.; Godt, A.; Jeschke, G. UWB DEER and RIDME Distance Measurements in Cu(II)-Cu(II) Spin Pairs. *J. Magn. Reson.* **2019,** *308*, 106560 https://doi.org/10.1016/j.jmr.2019.07.047.

(71) Collauto, A.; Frydman, V.; Lee, M. D.; Abdelkader, E. H.; Feintuch, A.; Swarbrick, J. D.; Graham, B.; Otting, G.; Goldfarb, D. RIDME Distance Measurements using Gd(III) Tags with a Narrow Central Transition. *Phys. Chem. Chem. Phys.* **2016,** *18*, 19037-49 https://doi.org/10.1039/c6cp03299k.

(72) Engelhard, D. M.; Meyer, A.; Berndhauser, A.; Schiemann, O.; Clever, G. H. Di-Copper(II) DNA G-Quadruplexes as EPR Distance Rulers. *Chem. Commun.* **2018,** *54*, 7455-7458 https://doi.org/10.1039/c8cc04053b.

(73) Keller, K.; Doll, A.; Qi, M.; Godt, A.; Jeschke, G.; Yulikov, M. Averaging of Nuclear Modulation Artefacts in RIDME Experiments. *J. Magn. Reson.* **2016,** *272*, 108-113 https://doi.org/10.1016/j.jmr.2016.09.016.

(74) Keller, K.; Mertens, V.; Qi, M.; Nalepa, A. I.; Godt, A.; Savitsky, A.; Jeschke, G.; Yulikov, M. Computing Distance Distributions from Dipolar Evolution Data with Overtones: RIDME Spectroscopy with Gd(III)-Based Spin Labels. *Phys. Chem. Chem. Phys.* **2017,** *19*, 17856-17876 https://doi.org/10.1039/c7cp01524k.

(75) Keller, K.; Qi, M.; Gmeiner, C.; Ritsch, I.; Godt, A.; Jeschke, G.; Savitsky, A.; Yulikov, M. Intermolecular Background Decay in RIDME Experiments. *Phys. Chem. Chem. Phys.* **2019,** *21*, 8228-8245 https://doi.org/10.1039/c8cp07815g.

(76) Keller, K.; Ritsch, I.; Hintz, H.; Hulsmann, M.; Qi, M.; Breitgoff, F. D.; Klose, D.; Polyhach, Y.; Yulikov, M.; Godt, A. et al. Accessing Distributions of Exchange and Dipolar Couplings in Stiff Molecular Rulers with Cu(II) Centres. *Phys. Chem. Chem. Phys.* **2020,** *22*, 21707-21730 https://doi.org/10.1039/d0cp03105d.

(77) Keller, K.; Zalibera, M.; Qi, M.; Koch, V.; Wegner, J.; Hintz, H.; Godt, A.; Jeschke, G.; Savitsky, A.; Yulikov, M. EPR Characterization of Mn(II) Complexes for Distance Determination with Pulsed Dipolar Spectroscopy. *Phys. Chem. Chem. Phys.* **2016,** *18*, 25120-25135 https://doi.org/10.1039/c6cp04884f.

(78) Razzaghi, S.; Qi, M.; Nalepa, A. I.; Godt, A.; Jeschke, G.; Savitsky, A.; Yulikov, M. RIDME Spectroscopy with Gd(III) Centers. *J. Phys. Chem. Lett.* **2014,** *5*, 3970-5 https://doi.org/10.1021/jz502129t.

(79) Stratmann, L. M.; Kutin, Y.; Kasanmascheff, M.; Clever, G. H. Precise Distance Measurements in DNA G-Quadruplex Dimers and Sandwich Complexes by Pulsed Dipolar EPR Spectroscopy. *Angew. Chem. Int. Ed.* **2020**, https://doi.org/10.1002/anie.202008618.
